# Spin-Pure Stochastic-CASSCF via GUGA-FCIQMC Applied to Iron–Sulfur Clusters

**DOI:** 10.1021/acs.jctc.1c00589

**Published:** 2021-09-01

**Authors:** Werner Dobrautz, Oskar Weser, Nikolay A. Bogdanov, Ali Alavi, Giovanni Li Manni

**Affiliations:** † Max Planck Institute for Solid State Research, Heisenbergstr. 1, 70569 Stuttgart, Germany; ‡ Yusuf Hamied Department of Chemistry, University of Cambridge, Lensfield Road, Cambridge CB2 1EW, United Kingdom

## Abstract

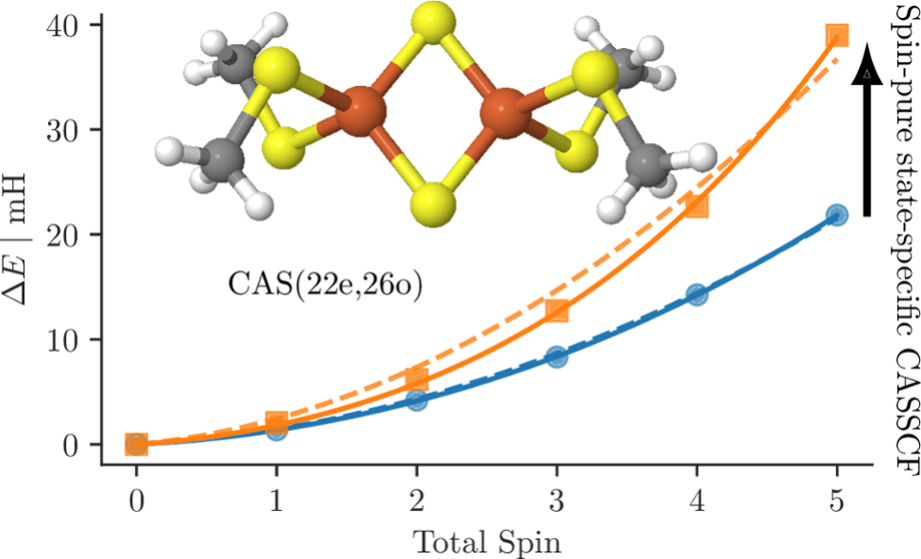

In this work, we demonstrate how to efficiently compute the one- and two-body reduced density matrices within the *spin-adapted* full configuration interaction quantum Monte Carlo (FCIQMC) method, which is based on the graphical unitary group approach (GUGA). This allows us to use GUGA-FCIQMC as a spin-pure configuration interaction (CI) eigensolver within the complete active space self-consistent field (CASSCF) procedure and hence to stochastically treat active spaces far larger than conventional CI solvers while variationally relaxing orbitals for specific spin-pure states. We apply the method to investigate the spin ladder in iron–sulfur dimer and tetramer model systems. We demonstrate the importance of the orbital relaxation by comparing the Heisenberg model magnetic coupling parameters from the CASSCF procedure to those from a CI-only (CASCI) procedure based on restricted open-shell Hartree–Fock orbitals. We show that the orbital relaxation differentially stabilizes the lower-spin states, thus enlarging the coupling parameters with respect to the values predicted by ignoring orbital relaxation effects. Moreover, we find that, while CASCI results are well fit by a simple bilinear Heisenberg Hamiltonian, the CASSCF eigenvalues exhibit deviations that necessitate the inclusion of biquadratic terms in the model Hamiltonian.

## 1 Introduction

The complete active space self-consistent field (CASSCF) method is a well-established approach in quantum chemistry for the treatment of strongly correlated electron systems with substantial multireference character.^1−8^ Important static correlation effects are rigorously described within the *active space*, consisting of the most important orbitals and electrons, while the effect of the *environment* (electrons not included in the active space) is accounted for at the mean-field level via a variational orbital optimization (the SCF procedure). One- and two-body reduced density matrices (1- and 2-RDMs) within the active space are necessary to perform orbital rotations between the active orbitals and the environment, whether a second-order Newton–Raphson formulation^2,7,9−11^ or the simplified super-CI technique with an average Fock operator is utilized.^4^ If applicable, exact diagonalization techniques^12−14^ are utilized to obtain eigenvalues, eigenvectors, and the RDMs associated with the CAS configuration interaction (CASCI) Hamiltonian. However, due to the exponential scaling of CASCI with respect to the size of the active space, exact diagonalization techniques are restricted to at most about 18 electrons in 18 orbitals, CAS(18e,18o), on a serial architecture.^15,16^ More recent massively parallel implementations allow sizes up to CAS(24e,24o).^17^ Another strategy is to use methods that approximate the full-CI wave function in the active space, like the density matrix renormalization group approach (DMRG),^18−30^ full configuration interaction quantum Monte Carlo (FCIQMC),^6,31−35^ selected configuration interaction (selected-CI) approaches^36−45^ (recently implemented in a spin-adapted form^46−48^), the correlation energy extrapolation by intrinsic scaling method,^49,50^ or the occupation restricted multiple active spaces (ORMAS),^51,52^ as well as the related generalized active space (GAS) approach,^53−55^ as CI eigensolvers within the CASSCF framework. However, while GAS was designed with the same GUGA framework discussed in the present work to enforce spin-adaptation,^7^ ORMAS is Slater-determinant-based and recently made use of the spin-flip configuration interaction method^56^ to ensure the correct spin multiplicity.^57^

These approaches allow the study of much larger active spaces.^6,39,40,55,58−64^ The use of FCIQMC as the CASSCF CI eigensolver within the super-CI framework, termed stochastic-CASSCF,^6^ has been developed in our group and used to study a number of strongly correlated systems, such as model systems of Fe(II)-porphyrins and the correlation mechanisms that differentially stabilize the intermediate spin states over the high-spin states,^55,58,60^ and model systems of corner-sharing cuprates.^59^ The original stochastic-CASSCF implementation was formulated using Slater-determinants (SDs) as the many-body basis for the CASCI wave function expansion. As SDs are not necessarily eigenfunctions of the total spin operator, their applicability is bound to the intrinsic spin structure of the system studied. If the low-spin states are energetically more stable and well separated from higher-spin states, it is possible to obtain essentially spin-pure wave functions when using an SD basis. However, when high-spin states are more stable than low-spin states, and/or a number of spin states are nearly degenerate, it is very difficult to obtain spin-pure solutions, or target states other than the ground state, with an SD basis.

In this paper, we present an algorithm for the calculation of 1- and 2-RDMs within the spin-adapted implementation of FCIQMC via the graphical unitary group approach (GUGA-FCIQMC).^65^ GUGA-FCIQMC has been implemented within the NECI code^31,66^ and provides accurate spin-adapted wave functions and RDMs for active space sizes out of reach for conventional exact CI eigensolvers.^62,63^ As already done for the original stochastic-CASSCF,^6^ the sampled 1- and 2-RDMs are then utilized within the super-CI procedure, as implemented in the OpenMolcas chemistry software package,^16^ to perform the orbital relaxation step. Thus, via the interface of the NECI code and OpenMolcas,^16^ it is possible to perform spin-adapted *state-specific* (or *state-average*, if RDMs of different states are weighted-averaged prior to the super-CI step) stochastic-CASSCF optimizations, targeting any desired spin state. The spin-pure stochastic-CASSCF allows us to obtain variationally optimized molecular orbitals, which in turn enable the calculation of spin gaps, unbiased from the choice of the starting orbitals.

The applicability and the importance of the method are shown through the investigation of the spin ladder of two iron–sulfur (FeS) clusters. Polynuclear transition-metal (PNTM) clusters are of major importance in organometallic chemistry and as cofactors in biology and are involved in a multitude of processes, including photosynthesis, respiration, and nitrogen fixation,^67−69^ being responsible for redox reactions^70−72^ and electron transfer,^73−81^ act as catalytic agents, and even provide a redox sensory function.^82^ A theoretical understanding of the intricate interplay of the energetically low-lying spin states of these systems, guided by accurate numerical results, could provide insights toward the synthetic realization of these processes. Especially, because direct experimental measurements targeting the electronic structures of these systems are often hindered by the large number of overlapping electronic states and corresponding vibrational modes at finite temperatures.^83−85^ In addition, some energetically low-lying excited states are inaccessible by accurate optical absorption experiments due to being electric-dipole-forbidden transitions.^86^

Spin-pure stochastic RDM sampling allows us to formulate a spin-adapted stochastic-CASSCF and gives us access to properties encoded in the 1- and 2-RDMs, such as spin–spin correlation functions. Using CASSCF wave functions of various active space sizes and compositions, we will study and discuss how spin gaps are affected by orbital relaxation effects. Additionally, the *ab initio* energies will be mapped to the (biquadratic) Heisenberg spin model^87−97^ to show the effect of active space size and orbital relaxation on the extracted magnetic coupling parameters, which are in turn compared to the available experimental data^98,99^ and other computational studies.^100,101^

The remainder of this paper is organized as follows: in section 2, we summarize the spin-adapted GUGA-FCIQMC method, and in section 3, we describe the sampling algorithm of spin-free RDMs. In section 4, we discuss *ab initio* CASSCF spin gaps and spin–spin correlation functions for an iron–sulfur dimer, Fe_2_S_2_,^62^ for different active space sizes and starting orbitals, and for an [Fe_4_S_4_] tetramer model system. We also map our *ab initio* results to a (biquadratic) Heisenberg model Hamiltonian, discuss the role of the CASSCF procedure when extracting the exchange parameter(s), and compare the magnetic coupling constants extracted from our computations to experimental and theoretical references. Finally, in section 5, we summarize our findings and offer a general discussion on the presented topic.

In Appendix A and Appendix C, we derive necessary formulas for local spin measurements and spin–spin correlation functions from RDMs, respectively. We additionally supply coordinate and orbital files, computational details, and comparisons with available exact results for small active spaces, a table with the data used in Figure 10, a study on improved convergence due to stochastic noise, the protocol on how we compared the orbitals in Figure 11, details on interface and the RDM storage convention in OpenMolcas, and a quick access literature overview of computational results for the Fe_2_S_2_ system in the Supporting Information (SI).

## 2 GUGA-FCIQMC

In this section, we briefly summarize the main details of the GUGA-FCIQMC implementation. More theoretical and technical aspects of the algorithm are available in the literature.^65,103^

The spin-adapted implementation of the FCIQMC algorithm relies on the unitary group approach (UGA),^104,105^ pioneered by Paldus, and its graphical extension (GUGA), introduced by Shavitt.^106,107^ GUGA provides an efficient-to-use spin-adapted many-body basis, based on the spin-free formulation of quantum chemistry.^108^ The spin-free form of the electronic Hamiltonian is given by
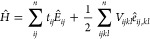
1with the spin-free excitation operators, *Ê*_*ij*_ = ∑_σ_*â*_*i*σ_^†^*â*_*j*σ_ and *ê*_*ij,kl*_ = *Ê*_*ij*_*Ê*_*kl*_ – δ_*jk*_*Ê*_*il*_, defined in terms of the creation and annihilation operators *â*_*i*σ_^†^, *â*_*j*σ_ with spatial orbitals *i*, *j*, and spin σ. *t*_*ij*_ and *V*_*ijkl*_ represent the one- and two-electron integrals in a molecular orbital basis, and *n* indicates the total number of spatial orbitals.

The name *unitary group* approach comes from the fact that the operators *Ê*_*ij*_ fulfill the same commutation relations as the generators of the unitary group of order *n*, *U*(*n*).^104^ Paldus^109,110^ identified a very efficient construction of a spin-adapted basis tailored for the electronic structure problem, based on the Gel’fand–Tsetlin basis,^111−113^ a general basis for any unitary group *U*(*n*). Paldus also demonstrated how to efficiently calculate Hamiltonian matrix elements, algebraically^104^ and graphically via the so-called “pattern calculus”,^109,110^ within this basis. Based on this seminal work, Shavitt developed the graphical extension of the UGA (GUGA),^106,107,114^ which provides an elegant and efficient way to calculate Hamiltonian matrix elements, ⟨ν|*Ĥ*|μ⟩, between different configuration state functions (CSFs), |μ⟩ and |ν⟩, within a chosen spin-symmetry sector, especially well suited to be combined with the FCIQMC method.

The first general purpose implementation of the GUGA was developed by Brooks and Schaefer,^115,116^ which is still available in the GAMESS*ab initio* quantum chemistry package.^117^ The combination of an efficient protocol for computing Hamiltonian matrix elements, and storage of the CI coefficients, enables an effective spin-adapted formulation of exact CI eigensolvers, such as CAS^1^ and GAS,^53^ perturbation theory methodologies, such as CASPT2,^118^ GASPT2,^119^ and SplitGAS,^120^ as well as the FCIQMC approach within the GUGA framework.^65^

The FCIQMC algorithm^33,34^ is based on the imaginary-time (τ = i*t*) Schrödinger equation
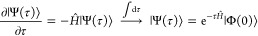
2which, after formal integration and a first-order Taylor expansion, yields an iterable expression for the eigenstate |Ψ(τ)⟩
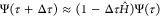
3FCIQMC stochastically samples the FCI wave function, |Ψ(τ)⟩, of a system by a set of so-called *walkers* and yields estimates for the ground- and excited-state^121^ energies and properties^122^ via the one- and two-body RDMs.^123^ Theoretical and algorithmic details on FCIQMC can be found in the literature,^33,34^ especially in the recently published review article ref (31).

At the heart of the FCIQMC algorithm is the so-called *spawning step*, which stochastically samples the off-diagonal contribution to the imaginary-time evolution of the targeted state
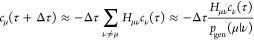
4with *c*_ν_(τ) being the coefficient of basis state function |ν⟩ at the imaginary-time τ, of the FCI expansion |Ψ(τ)⟩ = ∑_ν_*c*_ν_(τ)|ν⟩, and *p*_gen_(μ|ν) is the so-called *generation probability* of choosing configuration |μ⟩ given |ν⟩.

During an FCIQMC simulation, only coefficients that are at least occupied by a chosen minimum number of walkers (usually set to be the real number 1) are kept in memory. The off-diagonal contribution in eq 4 is then approximated by allowing each walker on each occupied configuration |ν⟩ to *spawn* new walkers on configuration |μ⟩ with a nonzero Hamiltonian matrix element ⟨μ|*Ĥ*|ν⟩. The process of suggesting a new configuration |μ⟩ given |ν⟩, called the *excitation generation step*, is of utmost importance.

**Figure 1:**
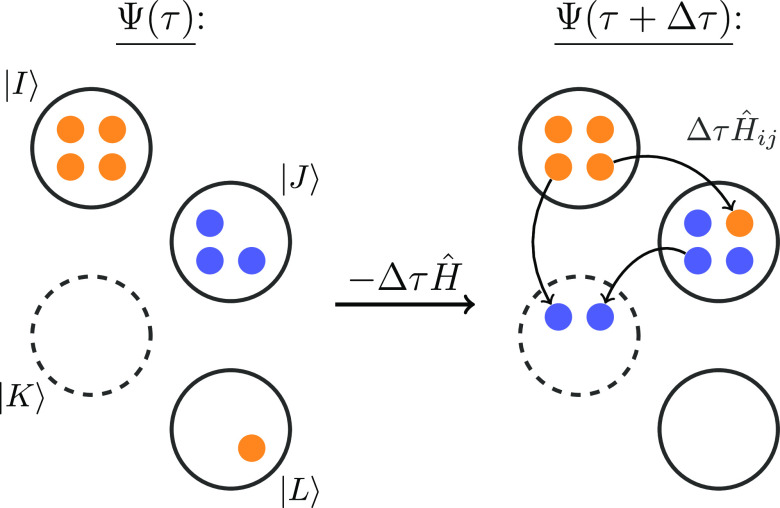
Schematic presentation of the FCIQMC spawning step. Orange and blue dots indicate opposite signed walkers on the stored basis states (black circles). Not stored states within a time-slice are indicated by dashed circles. The arrows point toward the newly spawned children after time Δτ has elapsed.

The maximal usable time step of the simulation is limited by the relation
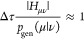
5to ensure stable dynamics. Hence, for large |*H*_μν_|/*p*_gen_(μ|ν) ratios, the time step of the calculation, Δτ, has to be lowered to ensure a stable simulation and is the motivation for optimizing the *excitation generation* step. Several schemes to obtain a close-to-optimal balance of computational effort and matrix element relation have been developed (see refs (31, 55, 124−126)). The spawning step is schematically shown in Figure 1.

**Figure 2:**
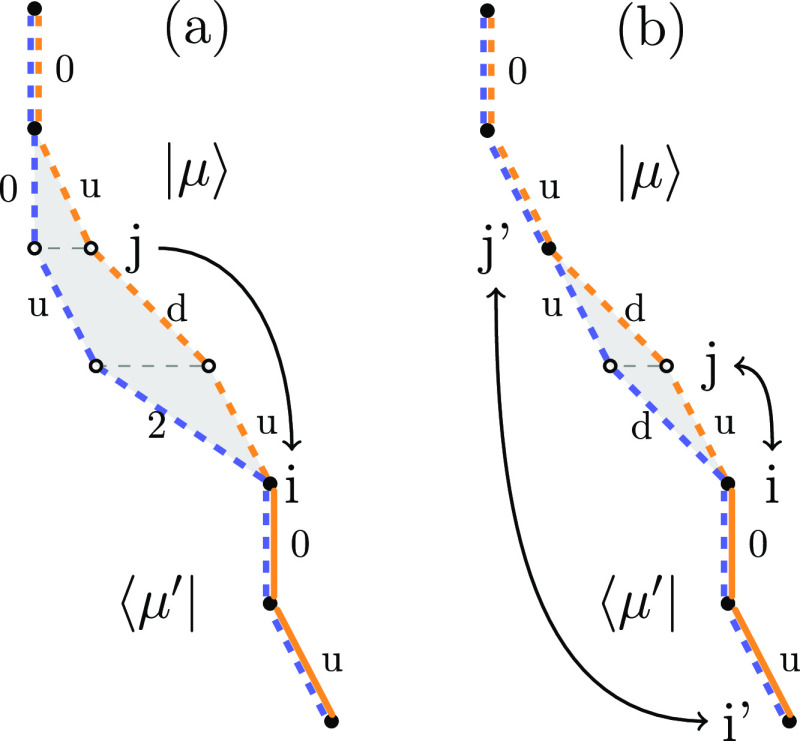
(a) Graphical representation of a possible single excitation from CSF |μ⟩ = |*u*, 0, *u*, *d*, *u*, 0⟩ to |μ′⟩ = |*u*, 0, 2, *u*, 0, 0⟩ by moving an electron from orbital *j* = 5 to *i* = 3 (indicated by the arrow on the left). The loop contributing to the coupling coefficient, ⟨μ|*Ê*_*ij*_|μ′⟩, is indicated by the gray area. Following Shavitt’s convention, the CSFs are drawn from bottom to top. (b) Exchange excitation example, for the same CSF |μ⟩, which shows that different index combinations for exchange excitations, *ê*_*ij,ji*_ and *ê*_*i*′*j*′,*j*′*i*′_, can lead to the same transition |μ⟩ → |μ′⟩ = |*u*, 0, *d*, *u*, *u*, 0⟩, with a nonzero coupling coefficient.

For reasons of interpretability, control, and improved convergence properties, a spin-adapted implementation of FCIQMC was long-sought after.^127^ GUGA allows an efficient spin-adapted FCIQMC implementation by constructing spin-symmetry-allowed excitations as stochastic walks on the graphical representation of CSFs, the so-called Shavitt graph, as depicted in Figure 2, and explained in more detail in refs (65, 103).

**Figure 3:**
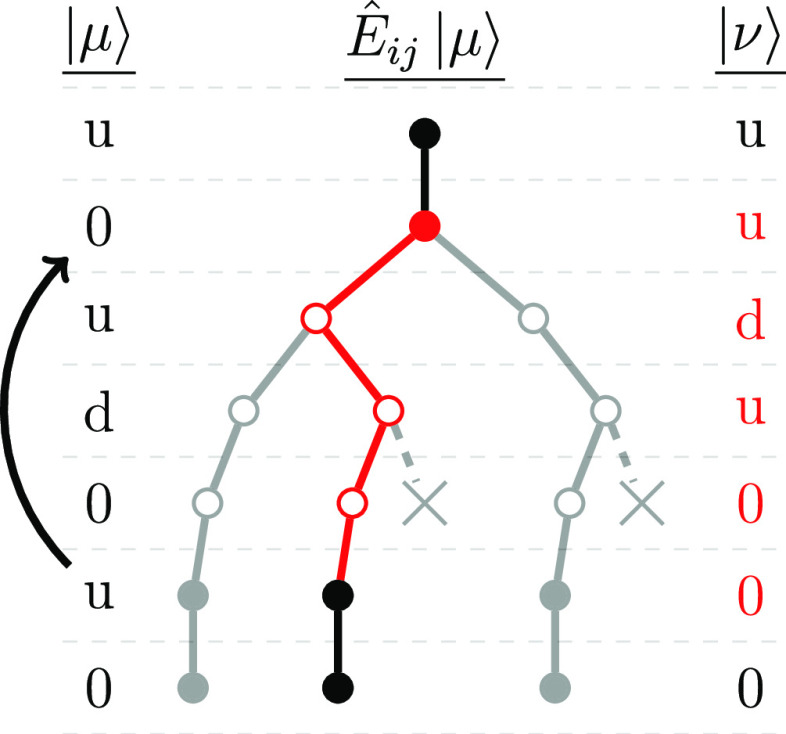
Schematic representation of the branching tree approach to allow efficient on-the-fly excitation generation and matrix element calculation entirely in the space of CSFs without any reference to SDs. An example is given for a single excitation *Ê*_*ij*_ from a CSF |μ⟩ = |*u*, 0, *u*, *d*, 0, *u*, 0⟩ to one other |ν⟩ = |*u*, *u*, *d*, *u*, 0, 0, 0⟩. In the shown example, an electron is excited from the singly occupied orbital 6 in |μ⟩ to the empty orbital 2 (indicated by the arrow on the left). Spin-allowed excitation pathways are indicated by solid lines. During the random excitation process in GUGA-FCIQMC, a spin-symmetry-allowed path is chosen at random, weighted according to the resulting coupling coefficient, ⟨μ|*E*_*ij*_|ν⟩ (indicated by the red pathway). In general, the empty starting orbitals and singly occupied orbitals in the excitation range allow for two possible spin couplings (*u*/*d*). Spin-symmetry-forbidden paths are indicated by the crossed-out nodes.

The GUGA allows both an efficient on-the-fly matrix element calculation and a way to select excitations from CSF |μ⟩ → |ν⟩ and ensures the approximate relation *p*_gen_(ν|μ) ∝ |*H*_μν_|, via a so-called *branching tree* approach. The stochastic GUGA excitation process for a single excitation, *Ê*_*ij*_, is schematically depicted in Figure 3, with the CSFs drawn from top to bottom. For a given CSF, |μ⟩, and two spatial orbitals, *i* and *j*, which are chosen with a probability weighted according to the magnitude of their *integral* contributions, at each open-shell orbital *k* within the range *i* → *j*, an allowed path is chosen randomly. This process is weighted with the so-called *probabilistic weight*, of the remaining decision tree below the current orbital *k*, which ensures the desired relation *p*_gen_(ν|μ) ∝ |*H*_μν_|. Interested readers are referred to refs (65, 103) for more details.

This stochastic process additionally circumvents the bottleneck given by the exponentially growing connectivity between CSFs with respect to the number of open-shell orbitals. Hence, our GUGA-FCIQMC method is able to treat systems with more than 30 open-shell orbitals, and due to the spin-pure formulation, it allows us to specifically target any spin-symmetry sector, removes any spin-contamination, reduces the Hilbert space size, and speeds up convergence in systems with near-degenerate spin states.^65,103^ However, compared to the SD-based FCIQMC, the calculation of (spin-free) RDMs in GUGA-FCIQMC is considerably more challenging, and hence such RDMs have not been available until now. This has prevented access to properties and using it as a spin-pure CI-solver within the stochastic-CASSCF method.^6^

## 3 GUGA-RDMs

In this section, the theoretical and algorithmic details of the stochastic sampling of RDMs within the GUGA-FCIQMC method are discussed.

### 3.1 Theoretical Considerations

Unbiased RDM sampling within the FCIQMC algorithm, whether in SD or CSF basis, is made possible by the *replica* method, where two independent dynamics are simultaneously carried out to remove a strictly positive bias due to stochastic fluctuations for the diagonal RDM contributions.^123^

In an SD-based implementation, the 1-particle RDM entries
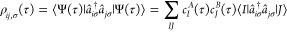
6are derived from the stochastic coefficients *c*_*I*_^*A*^(τ) and *c*_*J*_^*B*^(τ) of two statistically independent calculations, *A* and *B*. The two-body RDMs are obtained in a similar way. For SDs, the terms ⟨*I*|*a*_*i*σ_^†^*a*_*j*σ_|*J*⟩ are promptly given by the well-known Slater–Condon rules. We make use of the fact that |*I*⟩ → |*J*⟩ transitions are already performed in FCIQMC during the stochastic *spawning step*. Hence, we reuse the information, already required for a *normal* simulation, to additionally sample the 1- and 2-RDM elements.

In the original SD-based implementation, this is done by additionally storing information of the *parent* SD, |*J*⟩, along with the *spawned* new SD, |*I*⟩, including the parent SD encoded in a bit representation, its coefficient, in what run (*A* or *B*) this spawn happened, and other implementation-specific flags.

In a parallel high-performance computing (HPC) environment, the occupied determinants are distributed among the different processors. Hence, the newly spawned walkers are kept in an array, which has to be communicated to the corresponding processor, where the newly spawned state is stored, to update the corresponding coefficients.

The spin-free one- and two-body RDMs in terms of unitary group generators^128^ are defined as (following the convention of Helgaker, Jørgensen, and Olsen^3^)
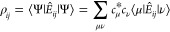
7with *Ê*_*ij*_^†^ = *Ê*_*ji*_, and
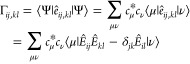
8with *i*, *j*, *k*, and *l* denoting *spatial* orbitals, |μ⟩ and |ν⟩ being CSFs, and *c*_μ_ and *c*_ν_ being their coefficients in the ground-state wave function expansion, |Ψ⟩.

The diagonal terms of the RDMs are accumulated *explicitly*, and the diagonal 1-RDM terms reduce to
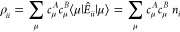
9where *n*_*i*_ is the occupation of the *spatial* orbital *i*, which can assume the values 0, 1, or 2. The diagonal 2-RDM elements are defined as
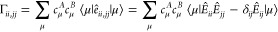
10which for *i* = *j* yields
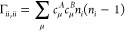
11and for *i* ≠ *j*
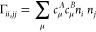
12eqs 11 and 12 are simply products of orbital occupation numbers and the coefficients *c*_μ_^*A*/*B*^ from two statistically independent simulations due to the above-mentioned positive bias in diagonal RDM entries. Exchange-type elements of the 2-RDM, Γ_*ij*,*ji*_, also have diagonal contributions from the wave function
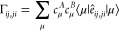
13which are also sampled explicitly. The detailed form of the coupling coefficients can be found in the literature.^65,107^ These exchange-like terms do, however, also have off-diagonal contributions, ⟨μ|*ê*_*ij,ji*_|ν⟩, which are explained in general in the following section.

### 3.2 Off-Diagonal RDM Entries: Computational Implementation and Cost

Similar to the SD-based RDM sampling, for each sampled RDM element, we store the parent state, |μ⟩, its coefficient, *c*_μ_, and the replica index (*A* or *B*). However, there are some important differences in the GUGA-based RDM sampling compared to those of an SD-based implementation:

(a) The one-electron *coupling coefficients*, ⟨μ′|*Ê*_*ij*_|μ⟩, and the corresponding two-body terms, ⟨μ′|*e*_*ij,ji*_|μ⟩, do not follow the Slater–Condon rules as for SDs. Shavitt and Paldus derived an efficient *product* form of these coupling coefficients, exemplified by a single excitation as
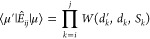
14where *W* is a function of the *step-values*, *d*_*k*_ = {0, *u*, *d*, 2}, of the spatial orbital *k* of the step-vector representation of the two CSFs, |μ⟩ and |μ′⟩, and the intermediate value of the total spin, *S*_*k*_, in the cumulative sense. The step-values, *d*_*k*_, encode if a spatial orbital is empty, *d*_*k*_ = 0, positively spin-coupled Δ*S*_*k*_ = +1/2, *d*_*k*_ = *u*, negatively spin-coupled, *d*_*k*_ = *d*, or doubly occupied, *d*_*k*_ = 2. CSFs can be represented graphically (see Figure 2a), where different step-values are indicated by a different tilt of the segments, and Shavitt showed that the value of the coupling coefficients only depends on the *loop shape* enclosed by the two coupled CSFs.
  Their explicit calculation scales with the number of spatial orbital indices between *i* and *j*. However, we calculate this quantity on-the-fly, during the excitation generation step, and thus, we can reuse it with no additional computational cost in the stochastic RDM sampling.
(b) *Identifying* the type of excitation and the involved *spatial* orbitals (*i*, *j*, *k*, *l*), when coupling CSFs, is a more complex operation than for SDs. CSFs can also differ in the open-shell spin coupling and not only in the specific spatial orbitals (*i*, *j*, *k*, *l*), yet still have a nonzero coupling coefficient. For example, in Figure 2a, we show Shavitt’s graphical representation of the CSF |μ⟩ = |*u*, 0, *u*, *d*, *u*, 0⟩ as the orange solid line and an excited CSF |μ′⟩ = |*u*, 0, 2, *u*, 0, 0⟩ as the blue dashed line. Following Shavitt’s convention, the CSFs are drawn from bottom to top. Only the gray *loop* area enclosed by both CSFs contributes to the coupling coefficient, ⟨μ|*E*_*ij*_|μ′⟩. The two CSFs are connected by an excitation of an electron from orbital *j* = 5 to *i* = 3, indicated by the arrow. However, as one can see in Figure 2a, the two CSFs |μ⟩ and |μ′⟩ do also differ in the spin coupling of orbital *k* = 4 with *d*_*k*_ = *d*, while *d*_*k*_^′^ = *u* (in the step-value notation). Hence, it is not as simple as performing bitwise logical operations on α- and β-strings as it is possible for SDs^129^ to identify the involved spatial indices and type of excitation. We do have optimized routines to perform this excitation identification for arbitrary CSFs in our GUGA-FCIQMC code NECI,^31,66^ and similar to the above-mentioned coupling coefficients, we already have the necessary information in the excitation process, within the spawning step.
(c) Certain excitation types, such as the exchange-like excitations, *ê*_*ij,ji*_ and *ê*_*ij,jk*_, can have multiple nonunique spatial orbital combinations leading to the same type of excitation |μ⟩ → |μ′⟩. This stems from the fact that certain contributions to the two-body coupling coefficients, ⟨μ|*ê*_*ij,kl*_|μ′⟩, are nonzero for alike open-shell step-values, *d*_o_ = *d*_o_^′^, above and below the loop spawned between |μ⟩ and |μ′⟩ (see Figure 2b and Shavitt).^107^

For example, for a pure exchange-type excitation, *ê*_*ij,ji*_, as depicted in Figure 2b, only the spin coupling of the open-shell orbitals differs, but there is no change in the orbital occupation. To calculate the Hamiltonian matrix element, ⟨μ|*Ĥ*|μ′⟩ = ∑_*i*≠*j*_*V*_*ijji*_⟨μ|*ê*_*ij,ji*_|μ′⟩, one needs to consider all nonzero contribution to the coupling coefficient, from orbital *i*′ below and *j*′ above the loop. Additionally, as the specific spatial orbitals, *i*, *j* (*k*, *l*) are chosen *first* in the excitation generation in FCIQMC, it is necessary to also take into account the possibilities that the other contributing orbitals, *p*(*i*′, *j*′), would have been picked (as their choice could have led to the same excitations) to assign a *unique* total generation probability, *p*_gen_(μ′|μ). However, for a correct RDM sampling, we have to retain the *original* probability *p*(μ → μ′|*i*, *j*, *k*, *l*) to sample a specific Γ_*ij,kl*_ entry to avoid a possible double counting. Conveniently, similar to the cases (a) and (b) mentioned above, we already have access to this specific quantity, obtained during the excitation generation process, and do not need to explicitly recalculate it for the stochastic RDM sampling.

The *three* additional necessary quantities discussed above, namely, the coupling coefficient, ⟨μ′|*Ê*_*ij*_|μ⟩ or ⟨μ′|*ê*_*ij,kl*_|μ⟩, the excitation type, and the probability *p*(μ → μ′|*i*, *j*, (*k*, *l*)), are already computed in the random excitation process. Consequently, the main change to enable spin-free RDM sampling within GUGA-FCIQMC is to communicate these three additional quantities, along with the already communicated information of the parent state, |μ⟩, its coefficient, *c*_μ_, and the replica index, *A*/*B*.

An important algorithmic advancement and routinely used feature of FCIQMC is the *semistochastic* method,^130,131^ where some chosen part of the Hilbert space, usually the *N*_D_ most occupied states, is treated explicitly. This is achieved by constructing the full Hamiltonian matrix *H*_μν_, ∀μ, ν ∈ {*N*_D_} and performing the imaginary-time evolution exactly. This necessitates also a change in the RDM sampling since the RDM contributions from states within the semistochastic space are not covered in the random excitation process anymore. These RDM contributions are treated exactly, greatly increasing their accuracy, but on the other hand, especially in the spin-free case, also increasing the computational effort. In this case, it is not possible to avoid the explicit *excitation identification* and *coupling coefficient* and *original generation probability calculation* in GUGA-FCIQMC. However, there is only a marginal computational overhead of around 10–20% associated with the spin-free RDM sampling compared to a standard two-replica FCIQMC calculation (see the SI for details).

The spin-adapted stochastic-CASSCF method has been made available in the OpenMolcas chemistry software package.^16^

## 4 Results and Discussion

**Figure 4:**
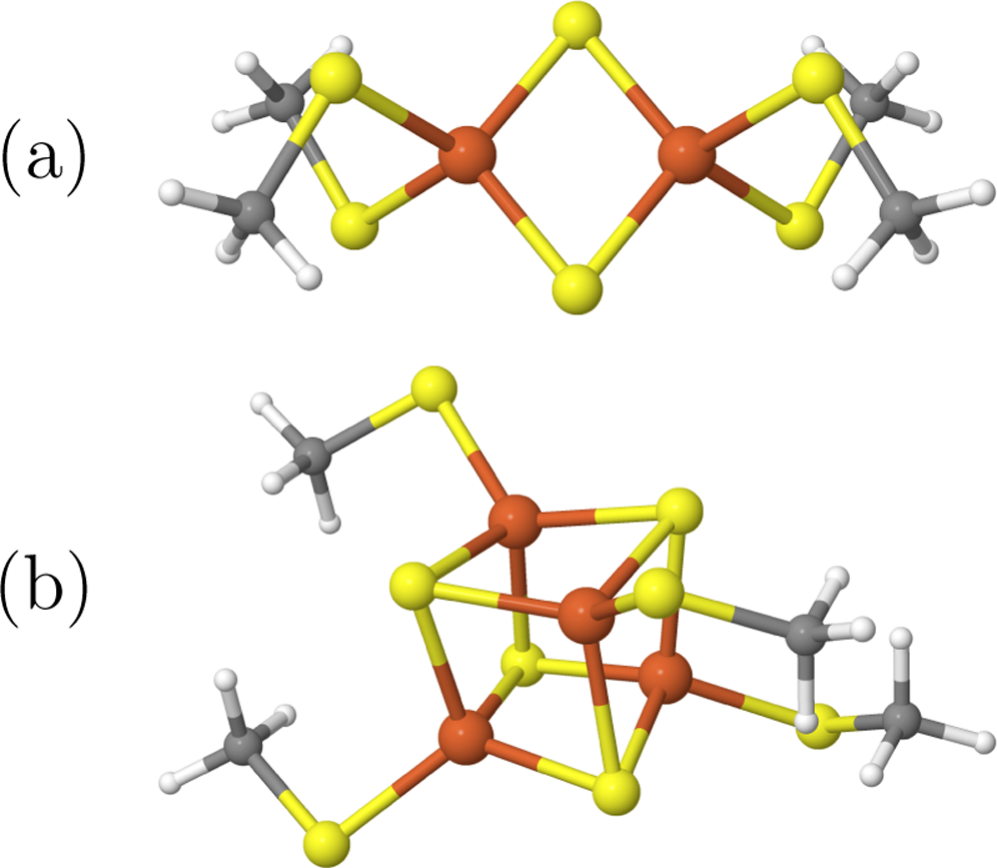
Geometry of the (a) [Fe_2_S_2_(SCH_3_)_4_]^2–^ model system derived from synthetic complexes of Mayerle et al.^132,133^ and (b) [Fe_4_S_4_(SCH_3_)_4_] model system obtained from synthetic complexes of Averill et al.^134^ Orange indicates iron, yellow indicates sulfur, gray indicates carbon, and white indicates hydrogen atoms.

The GUGA-FCIQMC RDM sampling has been used within the stochastic-CASSCF framework to study the low-energy spin states of the [Fe(III)_2_S_2_(SCH_3_)_4_]^2–^ model complex (Figure 4a), derived from synthetic complexes of Mayerle et al.,^132,133^ and utilized in our previous investigation,^62^ and the [Fe(III)_4_S_4_(SCH_3_)_4_] model cubane (Figure 4b), obtained from the synthetic complex of Averill et al.,^134^ where the terminal groups have been replaced by methyl groups. For the [Fe_2_S_2_] system, we considered (1) a CAS(10e,10o), consisting of the singly occupied iron 3d orbitals, (2) a CAS(10e,20o), consisting of the singly occupied iron 3d and the empty correlating double-shell d′ orbitals, (3) a CAS(22e,16o) consisting of the singly occupied iron 3d and the six doubly occupied bridging-sulfur 3p orbitals, and (4) a CAS(22e,26o) containing the iron 3d and d′ orbitals and the six bridging-sulfur 3p orbitals. This latter active space corresponds to that utilized in our previous work.^62^

We also studied the role of the iron 4s and the peripheral sulfur 3p orbitals, which were considered in other studies of similar FeS dimers,^100,135,136^ having mixed-valence states as the main target. We found that the iron 4s orbitals have a negligible differential role on the low-energy spin gaps.

Including one terminal orbital per peripheral sulfur atom in the active space resulted in an uneven mixing between different orbitals on some of the peripheral sulfur atoms upon completion of the CASSCF procedure. This suggests that for a balanced treatment of the peripheral S orbitals, one would need to include all 12 of them. However, while these orbitals have important ligand-field effects that could affect the energetic of mixed-valence states, we found that their role is less crucial for dealing with the homovalent [Fe(III)S] systems. Additionally, a recent study on the excited-state spectrum of the [FeS] dimer^137^ using the CAS(22e,16o) wave functions showed that the low-lying non-Hund excited states involve bridging-sulfur charge-transfer (CT) states, while CT states involving terminal-sulfur orbitals were only found at higher energies.

Thus, we decided not to further consider these orbitals in the chosen model active space. This was considered to be a successful strategy in previous works.^62,63,138^ Similar to previous computational studies,^62,63,100,135,136,138^ we do not include *empty* sulfur orbitals in our active space. Therefore, metal-to-ligand charge-transfer (MLCT) excitations are not considered by the model active space chosen. However, as also suggested by Neese et al.,^136^ such configurations are rather high in energy, and they can be safely neglected for low-energy spectrum calculations.

We used an extended relativistic atomic natural orbital basis of double-ζ quality for Fe atoms and a minimal basis for all other elements.^174^ The exactly diagonalizable Fe_2_S_2_ (10e,10o), (10e,20o), and (22e,16o) active spaces are straightforwardly calculable within spin-adapted stochastic-CASSCF with modest computational resources. We ensured the convergence of the (22e,26o) active space calculations with respect to the number of walkers, *N*_w_, by increasing *N*_w_ up to *N*_w_ = 1 × 10^9^ (see the SI for more information). The average number of occupied CSFs, *N*_CSF_, at each time step during the GUGA-FCIQMC calculation for *N*_w_ = 5 × 10^8^ and each spin state is shown in Table 1. The size of the deterministic space, *N*_D_, which is treated exactly within GUGA-FCIQMC, was *N*_D_ = 5 × 10^4^ for these calculations. With the spin-adapted implementation of FCIQMC via the GUGA, wave functions containing hundreds of millions of CSFs (with many open-shell orbitals) can be efficiently treated. Detailed further information on the geometries, orbitals, and computations can be found in the SI.

**Table 1:** Average Number of Occupied CSFs, *N*_CSF_ (in Millions), for Each Spin State in the Fe_2_S_2_ (22e,26o) Active Space Calculations with *N*_w_ = 5 × 10^8^

total spin	0	1	2	3	4	5
*N*_CSF_/10^6^	333	345	338	324	300	268

### 4.1 Fe_2_S_2_ System

The (10e,10o), (10e,20o), and (22e,16o) active spaces are exactly diagonalizable and were considered to study the differential interplay of different correlation mechanisms, such as orbital relaxation, *double-shell,*(58,139−141) and superexchange^59,142−145^ correlation effects, and to benchmark and test our stochastic spin-free RDM sampling procedure. A thorough comparison of the exact and the stochastic-CASSCF results can be found in the SI.

In our earlier works,^62,63^ we have demonstrated via theoretical arguments, and shown with calculations, that the choice of orbital representation and reordering greatly affect the sparsity of the CI wave function within the GUGA formalism. We have also shown that the localization and reordering strategy within the GUGA-FCIQMC algorithm is of utmost importance, as it positively influences the stability of the dynamics and the convergence with respect to the total number of walkers. Moreover, this strategy greatly simplifies the interpretation of the converged wave functions and could even allow selective optimization of one among ground- and low-energy excited-state wave functions. We have adopted the same strategy for the present work. In ref (62), the optimized CASSCF(22e,26o) orbitals for the *S* = 0 ground state, obtained via the SD-based stochastic-CASSCF,^6^ were used as starting orbitals for the localization and reordering protocol and for the GUGA-FCIQMC dynamics. A CASSCF(10e,10o) was performed inside the CAS(22e,26o) active space, an invariant rotation within the CAS(22e,26o), that separates valence 3d orbitals from the six sulfur and the 10 correlating d′ orbitals. Only the 10 valence 3d orbitals were localized and site-ordered, leaving the sulfur and the correlating d′ orbitals delocalized. In the present work, the starting orbitals were obtained from a high-spin restricted open-shell Hartree–Fock (ROHF) calculation, equivalent to a CASSCF(10e,10o) *S* = 5 optimization. The iron 3d and d′ orbitals, resulting from the ROHF calculation, were separately localized, using the Pipek–Mezey^146^ method, while the bridging-sulfur 3p orbitals were left delocalized. Using localized d′ orbitals allows us to better estimate the local spin of each magnetic center.

#### 4.1.1 Fe_2_S_2_ Spin Ladder and Total Energies

**Figure 5:**
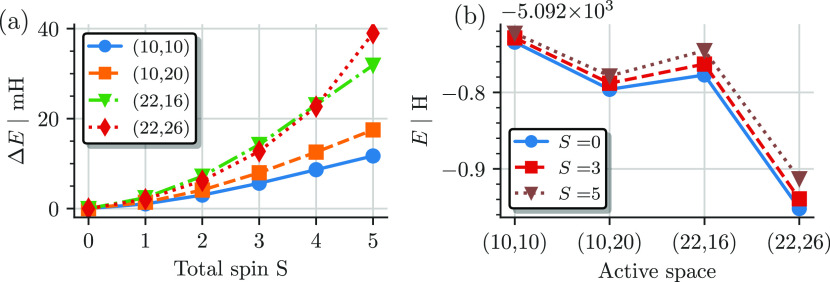
(a) CASSCF spin gaps relative to the *S* = 0 state as a function of the total spin *S* for different active spaces and (b) total CASSCF energies of the *S* = 0, 3, and 5 states as a function of the active spaces.

Figure 5a shows the spin gaps of all of the states relative to the *S* = 0 ground state, the *spin ladder*, as a function of the total spin after the CASSCF orbital optimization. The spin gaps are lowest in the (10e,10o) active space, with Δ*E* = 12 mH, between the *S* = 5 and *S* = 0 states. The inclusion of the iron d′ orbitals in the (10e,20o) active space qualitatively does not change the obtained spin ladder, and it also has a rather smaller quantitative effect, with only slightly larger Δ*E* = 17 mH between the *S* = 5 and *S* = 0 states. Inclusion of the bridging-sulfur 3p orbitals has the largest effect on the spin gaps since it accounts for the metal-bridging ligand correlation, which is differentially more important than the radial correlation effect,^140^ accounted for by the inclusion of the d′. The consideration of both the iron d′ and the bridging-sulfur 3p orbitals in the (22e,26o) active space induces a qualitative change in the obtained spin gaps, which will be further discussed below. Quantitatively, the relative spin gaps enlarge by as much as a factor of 3.3, when enlarging the active space, from CAS(10e,10o) to CAS(22e,26o).

In Figure 5b, we show the total energy of the *S* = 0, 3, and 5 states, helpful in describing in absolute terms the correlation effects bound to ligand-to-metal charge transfer and radial correlation effects. Starting from the CAS(10e,10o), the inclusion of the iron correlating d′ orbitals, as in the CAS(10e,20o) active space, lowers the total energy more than including the sulfur 3p orbitals, as in the CAS(22e,16o). The combined inclusion of both iron d′ and sulfur 3p orbitals has the surprising effect of lowering the total energies more than the ligand-to-metal charge transfer and the radial correlation effects on their own. However, the largest differential effect arises from the ligand-to-metal charge-transfer excitations as shown in Figure 5a.

#### 4.1.2 Orbital Relaxation Effect

In this section, the overall and the differential effects of the CASSCF orbital relaxation on energies and spin gaps, together with its effect on the derived model parameters, are discussed. The highest-spin, *S* = 5, ROHF orbitals from the (10e,10o) active space are chosen as starting orbitals for all of the calculations. The results of the first CASSCF iteration are from here on referred to as CASCI.

**Figure 6:**
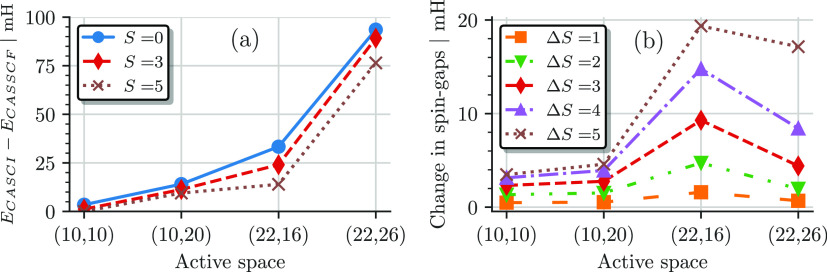
(a) Change of the total energy for the *S* = 0, 3, and 5 states and (b) change of the spin gaps relative to the *S* = 0 state due to the CASSCF orbital relaxation as a function of active space using (10e,10o) ROHF as starting orbitals (CASCI).

Figure 6a shows the energy difference of the CASCI results using (10e,10o) ROHF orbitals and the CASSCF results, Δ*E* = *E*_CASCI_ – *E*_CASSCF_, for the *S* = 0, 3, and 5 states as a function of the active space. As expected, the effect of the CASSCF orbital relaxation, when using (10e,10o) ROHF orbital, is lowest for the (10e,10o) active space (with differences below 10 mH) and highest for the (22e,26o) active space. Within each active space, the effect of the CASSCF procedure is largest for the low-spin states, with a maximum difference of Δ*E* = 94 mH for the singlet in the (22e,26o) active space. For the high-spin states, the effect of the CASSCF procedure is smaller but still substantial for the larger active spaces, especially in the (22e,26o) active space (AS), with Δ*E* = 76 mH for the *S* = 5 state.

To investigate the differential effect, Figure 6b shows the changes in the spin gaps due to the CASSCF orbital relaxation as a function of active space. As expected, the CASSCF procedure increases all of the obtained spin gaps, as the low-spin states are stabilized more by the orbital relaxation when starting from high-spin ROHF orbitals than the higher-spin states, which are better represented by the ROHF orbitals. The change in the spin gaps is smallest for the (10e,10o), where the ROHF starting orbitals were obtained, and the somehow similar (10e,20o) active space. Interestingly, although the effect of the CASSCF procedure on the total energies is highest for the (22e,26o) active space (see Figure 6a), the largest effect on the spin gaps is observed in the intermediate (22e,16o) active space. The energy differences to low-spin states, Δ*S* = 1, 2, 3, are affected only weakly by the CASSCF optimization and stay similar to the CASCI results. This can be explained by the fact that the low-spin states are similarly biased in the ROHF orbital basis and thus show similar stabilization during the CASSCF procedure. The high-spin states, on the other hand, are less stabilized by the CASSCF procedure, and as a consequence, the low-to-high-spin gap results are enlarged by the orbital relaxation.

There is a substantial differential effect of ≈15–20 mH due to the CASSCF orbital relaxation, so one has to be cautious when using ROHF orbitals for spin systems, and orbital bias toward the high spin is to be expected, leading to a systematic underestimation of spin gap predictions for antiferromagnetically coupled magnetic sites. Even for the seemingly SCF-invariant singlet–triplet spin gap, the CASSCF procedure is crucial to obtain more accurate model magnetic parameters, as will be discussed below.

**Figure 7:**
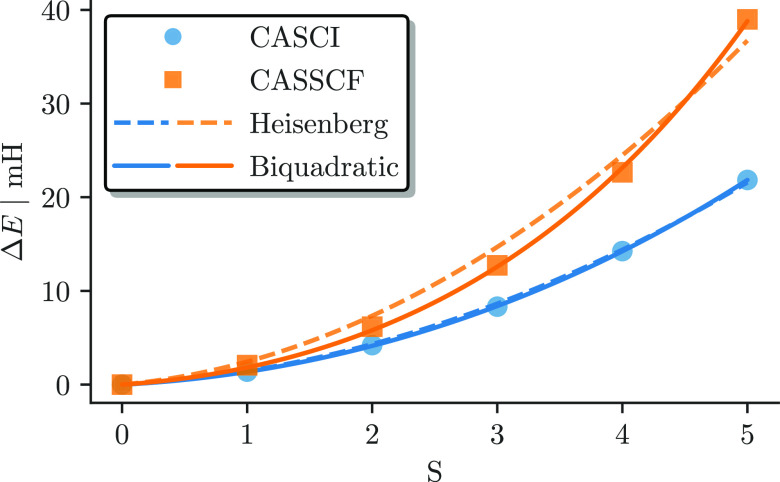
Energy difference to the *S* = 0 ground state as a function of spin in the (22e,26o) active space for the *ab initio* CASCI (blue) and CASSCF (orange) results with a simple (dashed line) and biquadratic (solid line) Heisenberg model fitted to the data.

Figure 7 shows the energy differences with respect to the *S* = 0 ground state for the CASCI(22e,26o) (blue circles) and for the CASSCF(22e,26o) (orange square) results. As expected, the spin states are more separated after the CASSCF orbital optimization, with the lowest-to-highest-spin-state gap nearly doubled by the orbital relaxation effects. Figure 7 also shows the spin ladder obtained from mapping the *ab initio* results to a Heisenberg model,^87−89,91,92^ without (dashed lines) and with (solid lines) a *biquadratic* correction. This aspect will be discussed in greater detail in the next section.

#### 4.1.3 Mapping to a Spin Model

As previously done by Sharma et al.^100^ and in our laboratories,^63^ we map the ab initio low-energy spectrum of the Fe_2_S_2_ system to a spin Hamiltonian, as the spin-exchange interactions are the dominant form of magnetic interactions in this system. First, we map the excitation energies of the Fe_2_S_2_ system to the *linear* two-site Heisenberg Hamiltonian
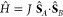
15with eigenvalues
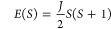
16where **Ŝ**_*A*/*B*_ are the local spin-^5^/_2_ operators of the two iron centers, and *S* is the total targeted spin. We obtain the magnetic coupling parameter *J* by performing a least-squares fit of the energy expression (eq 16) to the *ab initio* results of all lowest spin states and study the quality of this mapping as a function of the active space size and the effect of the CASSCF orbital optimization. As shown in Figure 7, the bilinear Heisenberg spin ladder (dashed blue line) models the *ab initio* CASSCI results with high accuracy. However, minor deviations can be observed for the fitting of the CASSCF results. This finding suggests that orbital relaxation effects account for additional forms of interactions between the metal centers in addition to enlarging the predicted *J* values. An improved Heisenberg model with *biquadratic* exchange^89−97^
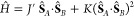
17with eigenvalues
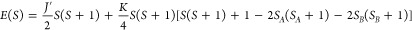
18greatly improves the fitting of the model Hamiltonian (solid lines in Figure 7).

**Figure 8:**
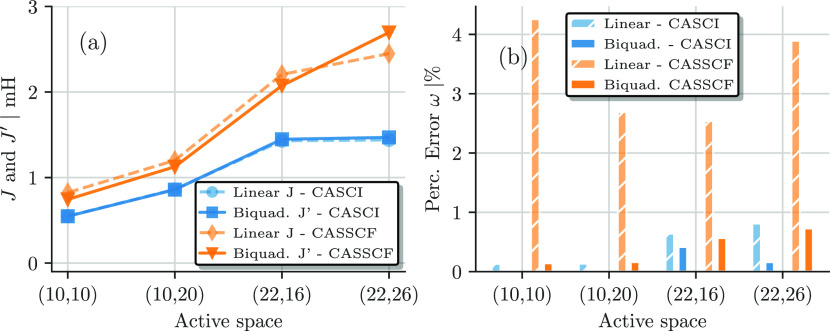
(a) Bilinear Heisenberg *J* (dashed lines) and biquadratic *J*′ (solid lines) fit of the *ab initio* CASCI (blue) and CASSCF (orange) results as a function of the active space sizes. (b) Relative average error per state ω in percent of the corresponding bilinear (dashed) and biquadratic (solid) Heisenberg fits of panel (a) as a function of the active space.

Figure 8a shows the fitted model parameters of the bilinear, *J* (eq 15), and biquadratic, *J*′ (eq 17), Heisenberg models as a function of the active space size for the CASCI (blue squares and circles) and CASSCF (orange triangles and diamonds) results. For the CASCI results (blue), the extracted model parameters, *J* and *J*′, are almost identical for all active spaces, indicating a good description by the bilinear Heisenberg model. *J* and *J*′ increase from a value of 0.55 mH in the (10e,10o) active space to about 1.44 mH in the (22e,16o) and (22e,26o) AS.

For the CASSCF results (orange), the extracted *J* (diamonds) and *J*′ (triangles) parameters are larger than the corresponding CASCI results, increasingly so in the larger active spaces, and additionally, the bilinear *J* and biquadratic *J*′ mildly differ. For all but the largest active space, the biquadratic *J*′ is about 0.1 mH smaller than the bilinear *J*, while it is ∼0.25 mH larger in the (22e,26o) AS. The differences between the extracted model parameters indicate that a simple bilinear Heisenberg model is not sufficient to describe the CASSCF results.

To quantify this discrepancy and analyze how well a biquadratic model suits the *ab initio* results, we show the relative average error per state ω (in percent) of the corresponding bilinear and biquadratic Heisenberg fits to the CASCI (blue solid and striped) and CASSCF (orange solid and striped) results in Figure 8b. Following ref (90), ω is defined as
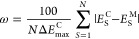
19where *E*_S_^C^ is the computed *ab initio* spin gap of spin state S relative to the singlet ground state and *E*_S_^M^ is the energy obtained by fitting the bilinear and biquadratic model (eqs 16 and 18). *N* is the number of considered states (with *N* = 5 in the FeS dimer case, as we only consider the spin gap relative to the singlet ground state), and Δ*E*_max_^C^ is the *ab initio* energy difference between the *S* = 5 and singlet states.

The CASCI spin ladders exhibit a clear bilinear Heisenberg behavior, as shown by the small ω values (blue bars) in Figure 8b. The error is less than 1% for all active space sizes. Larger discrepancies emerge between the CASSCF energies and the bilinear Heisenberg model, indicated by larger ω values (orange striped bars in Figure 8b). The relative error ω, defined in eq 19, takes into account the gap between the *S* = 0 and *S* = 5 states, Δ*E*_max_^C^, in the denominator. This causes ω to be largest for the bilinear Heisenberg fit to the CASSCF results in the (10e,10o) active space.

Overall, these discrepancies are still rather small (at most 4%), as shown in Figure 8b; however, they are not negligible. The biquadratic Heisenberg Hamiltonian (eq 17) describes the CASSCF results better, as indicated by a much smaller ω value (less than 1%) in all cases (see Figure 8b). However, the largest CAS(22e,26o) starts to show deviations also from the biquadratic Heisenberg model. Independently of the quantitative aspects, our calculations confirm the antiferromagnetic character of this system, with the CASSCF predicting a larger antiferromagnetic magnetic constant than the CASCI procedure. This result, although very promising, is not definitive, and in fact, correlation effects, not accounted for in the present work, such as dynamic correlation effects outside the active space and convergence with the basis set, could further enhance the deviation from the biquadratic Heisenberg Hamiltonian.

Considering the results of the present work and those available in the literature^98,100,101,136,138,147,148^ (see the SI for details), some clear trends can be promptly recognized: increasing the active space, performing CASSCF orbital optimization, and/or recovering dynamic correlation widens the energy spread of the spin ladder, and, thus, yielding a larger effective magnetic coupling coefficient *J*. The almost doubling of the extracted *J* and *J*′ due to the CASSCF procedure, as seen in Figures 7 and 8a, indicates the important role of orbital relaxation by differentially stabilizing the low-spin state.

This finding clearly shows that one needs to be cautious when using CI energies on ROHF orbitals, and a systematic error is to be expected that overstabilizes higher-spin states over low-spin states. Moreover, the deviation from the simple bilinear Heisenberg model, although small, indicates that the complexity of the interactions in [FeS] clusters cannot simply be reduced to a Heisenberg spin system when aiming at quantitative accuracy; instead, more involved forms of interactions are present, which require complex *ab initio* Hamiltonians (here exemplified by large CASSCF calculations) and model Hamiltonians (here exemplified by the biquadratic Heisenberg).

#### 4.1.4 CASSCF Effects on Local Spin Measurements for Fe_2_S_2_

To further investigate the applicability of a (biquadratic) Heisenberg spin model, we look into local spin measurements and spin–spin correlation functions between the two iron centers and study the CASSCF effect on these quantities. We explain in Appendix A how we directly measure these quantities, and in Appendix C and Appendix D we explain how to extract them from the spin-free 1- and 2-RDMs. We want to emphasize that we are aware that the local spin and spin–spin correlation functions between single and sums of orbitals are representation-dependent quantities, meaning they are not actual physical observables but do depend on the type of employed orbitals, i.e., localized or delocalized orbitals. However, they are still extremely useful means to provide insight into the chemical and physical properties of compounds and accordingly are extensively used in the literature.^100,149−151^

To ensure reproducibility of our results, we want to point out the protocol to obtain the orbitals we used again: the starting orbitals for all calculations were the (10e,10o) ROHF orbitals, for which the iron 3d and 3d′ were identified and separately localized with the default options of the Pipek–Mezey^146^ method in OpenMolcas.(15,16) These orbitals were then relaxed during the stochastic-CASSCF procedure, and the converged orbitals, which remained very localized and in the initial atom-separated order (discussed further below), were used to obtain the corresponding local spin and spin–spin correlation functions. We tested the stability of these results by (a) localizing the final CASSCF orbitals and (b) performing a Procrustes^55,152^ transformation to map the starting ROHF orbitals as close as possible to the CASSCF orbitals and found no effect on the obtained local spin and spin–spin correlation functions.

**Figure 9:**
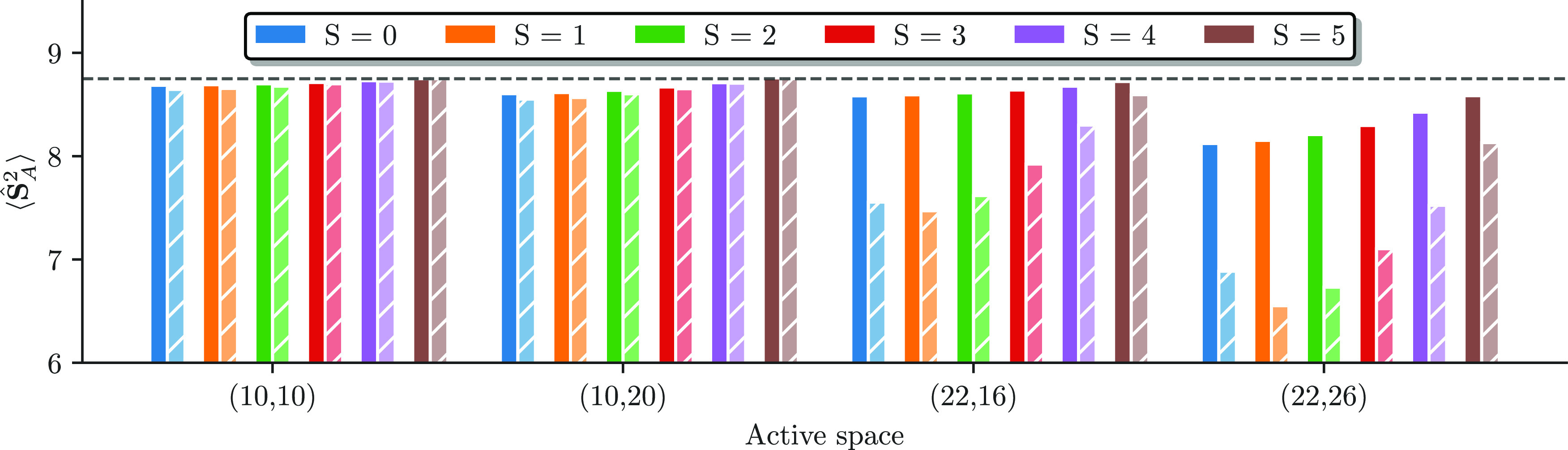
Local spin expectation value on iron *A*, ⟨**Ŝ**_*A*_^2^⟩, from the CASCI (solid bars) and the final CASSCF results (striped bars) as a function of the active space size for all spin states. The dashed line indicates the maximal possible value of 8.75.

Figure 9 shows the local spin expectation value on iron *A*, ⟨**Ŝ**_*A*_^2^⟩, extracted from the CASCI (solid bars), and the CASSCF wave functions (striped bars), for different active spaces and all accessible spin states. The CAS(10e,10o) and CAS(10e,20o) exhibit a local spin expectation value close to the maximum possible, , for all spin states. The CASSCF orbital relaxation does not have a significant impact on it. Upon inclusion of the bridging-sulfur orbitals in the CAS(22e,16o), enabling ligand-to-metal (“superexchange-type”) excitations, the local spin expectation value remains close to the maximum for CASCI results. However, it substantially drops for all spin states upon CASSCF orbital relaxation. This behavior is enhanced for the CAS(22e,26o); however, for this choice of active space, a reduced local spin expectation value for the low-spin states is already obtained for the CASCI calculations. Interestingly, the triplet in the CAS(22e,16o) and CAS(22e,26o) and the quintet in the CAS(22e,26o) have a lower local spin expectation value than the singlet after the CASSCF procedure.

The CASSCF local spin expectation value of the triplet state in the CAS(22e,26o) of ⟨**Ŝ**_*A*_^2^⟩_min_ ≈ 6.5 corresponds to a local spin of *S*_*A*_ ≈ 2, which raises the question of the applicability of the Heisenberg model mapping. In general, for systems with local spin momenta larger than *S* = ^1^/_2_, local non-Hund excited states can cause deviations from a pure Heisenberg behavior.^89,92,96,153,154^ Additionally, as discussed by Sharma et al.,^100^ the deviation from the pure *S* = ^5^/_2_ ion demands accounting for spin and charge delocalization. Both contributions can be related to additional biquadratic terms in the Heisenberg Hamiltonian.^90−92,153^

**Figure 10:**
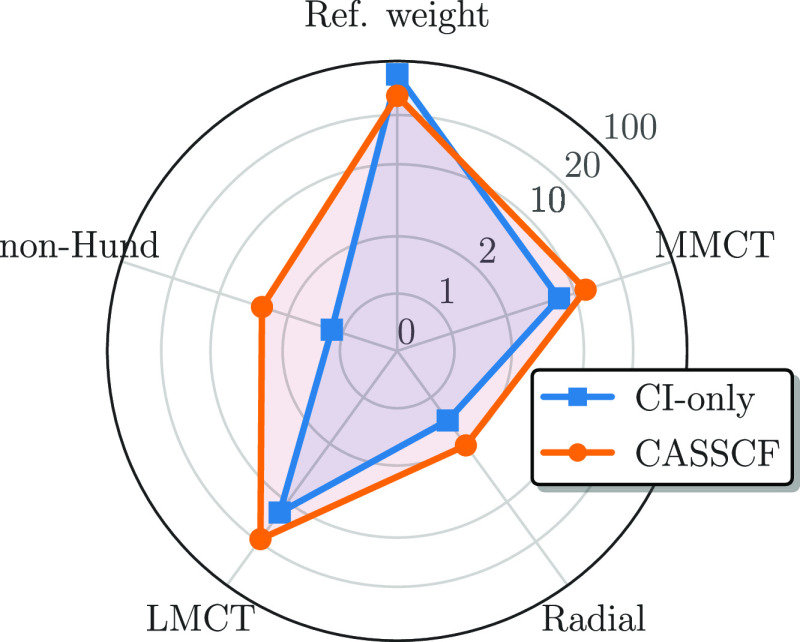
Radar plot showing the most important contributions to the CASCI (blue squares) and CASSCF (orange circles) singlet ground state in the (22e,26o) active space in percent. The figure shows the reference weight (ref weight), inter-iron 3d ↔ 3d charge transfer (MMCT), intra-iron “breathing”-like 3d → d′ radial (Radial), bridging-sulfur-to-metal CT (LMCT), and local Hund’s rule-violating intra-iron 3d → 3d excitations (non-Hund).

One striking advantage of our methodology, based on the FCIQMC algorithm applied onto localized and site-ordered MOs, is that we have direct access to the stochastic representation of the ground-state wave function. Thus, to further analyze the deviations from a pure Heisenberg model, we investigated the leading contributions to the CASCI and CASSCF results for each studied active space. As an example, we show in a radar plot (Figure 10) the reference weight (ref weight), the sum of all metal-to-metal charge transfer (MMCT), local d → d′ radial excited configurations (Radial), ligand-to-metal charge transfer (LMCT), and local Hund’s rule-violating configurations (non-Hund) for the CASCI (blue squares) and CASSCF (orange circles) singlet state in the (22e,26o) active space. It is important to note that the values are displayed in percent, and the radial axes (indicated by the above-introduced acronyms) are on a logarithmic scale to allow an easier visual comparison of the different contributions to the ground-state wave function, and the explicit values can be found in the SI.

The reference weight of the *S* = 0 state in the (22e,26o) active space drops from a value of 74.4% in the CASCI to 46.1% in the CASSCF wave function. On the other hand, the inter-iron MMCT (Fe_*A*_ 3d ↔ Fe_*B*_ 3d) increases from 6.9% to 12.9% and the bridging-sulfur-to-metal LMCT increases from an already large 13.4% to a substantial 27.9% between the CASCI and CASSCF calculations. Both the radial-type, intra-iron 3d → 3d′ (CASCI: 1.5%, CASSCF: 2.1%) and intra-iron non-Hund configurations (CASCI: 1.2%, CASSCF: 3.7%) only have marginal contributions in the wave functions. The remaining spin states show similarly large LMCT contributions after the CASSCF procedure in the (22e,26o) active space.

These results suggest that the main driving forces in lowering the local spin expectation values are LMCT configurations upon inclusion of the bridging-sulfur orbitals in the active space. However, as shown in Figure 9 by the relatively constant (close-to-maximum) CASCI local spin expectation values for all active space, “just” including the sulfur 3p orbitals does not suffice to correctly capture all relevant correlation mechanisms; instead, the CASSCF orbital relaxation of the ROHF starting orbitals is necessary.

Malrieu et al.,^155,156^ Angeli and Calzado,^157^ and Li Manni and Alavi^58^ have observed that CASSCF orbitals from a minimal active space are *too localized* to correctly capture all relevant physical mechanisms in a subsequent second-order multireference perturbation theory (MRPT2). This is mainly due to the fact that relevant ligand-to-metal charge-transfer (LMCT) excitations do not interact with the zeroth-order wave function due to the generalized Brillouin theorem.^158−160^ On the other hand, natural magnetic orbitals, obtained by, e.g., difference-dedicated CI (DDCI) calculations,^161−165^ or optimized CASSCF orbitals from large active space calculations,^58−60^ show *correlation-induced* metal–ligand delocalization by capturing higher-order contributions.^58,155,156^

We also studied this effect in the present work by directly comparing the localized high-spin *S* = 5 (10e,10o) ROHF orbitals (used as the starting orbitals in all CASSCF calculations) with the singlet (22e,26o) CASSCF orbitals. During the stochastic-CASSCF procedure, performed with OpenMolcas, the orbitals remain quite localized and in the chosen atom-separated order, mentioned above and described in the SI. For reproducibility, it is important to note that we used the last orbitals of the OpenMolcas CASSCF procedure *before* the standard final diagonalization of the 1-RDM and transformation to natural (delocalized) orbitals. Furthermore, we performed invariant Procrustes orthogonal transformations,^55,152^ with the OpenMolcas software package, of the (10e,10o) ROHF iron 3d orbitals to make them as similar as possible to the (22e,26o) singlet CASSCF orbitals to allow an optimal comparison. Further details of the exact protocol for the comparison and corresponding orbital files can be found in the SI.

**Figure 11:**
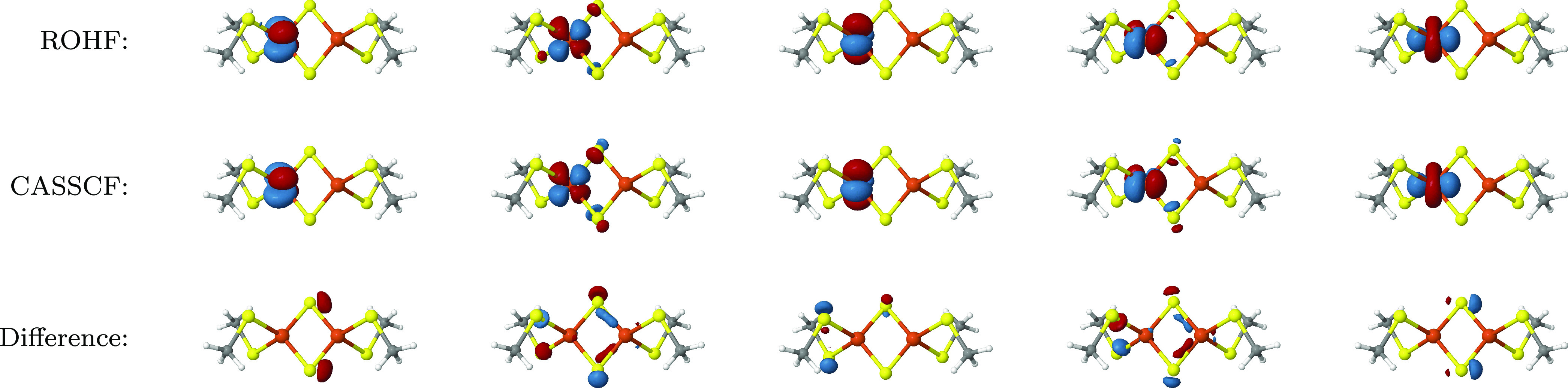
(10e,10o) ROHF (top row) and (22e,26o) *S* = 0 CASSCF Fe_*A*_ 3d orbitals rendered with Jmol(166) with an isosurface value of 0.05. The difference between the corresponding ROHF and CASSCF orbitals (bottom row) was obtained with Multiwfn(168) and is rendered with an isosurface cutoff of 0.007 (except the 3rd column, which uses a value of 0.003). The protocol to obtain the orbitals and their differences is described in the main text and with more detail in the SI, where also the corresponding orbital files can be found.

In Figure 11, we show the (10e,10o) ROHF (top row) and the CASSCF(22e,26o) singlet (middle row) 3d orbitals of iron *A*, rendered with the Jmol software package,^166^ with an isosurface cutoff value of 0.05. The last row of Figure 11 shows the difference of the corresponding orbitals, computed with the pegamoid.py(167) and Multiwfn software packages^168^ and rendered with Jmol with an isosurface cutoff value of 0.007 for all orbitals except the third (3rd column), which has a cutoff value of 0.003 to make differences visible. The delocalization effect of the CASSCF procedure can be seen for orbitals two (2nd column) and four (4th column). The orbital differences show that the CASSCF procedure has a metal-to-ligand delocalization effect, where larger tails of the iron 3d orbitals on the ligands increase both the kinetic and direct exchange integrals^143,169^ and consequently increasing the absolute value of *J*.^156,157^ As discussed above and shown in Figure 10, the delocalization of the iron 3d orbitals is accompanied by a simultaneous increase of the LMCT contributions in the (22e,26o) CASSCF singlet wave function.

Calzado et al.^156^ show a very similar orbital dependence when performing CASCI calculation on extracted *J* parameters. Their study on local *S* = 1 binuclear systems shows that the high-spin triplet ROHF orbitals yield a much too low *J* compared to using singlet or state-specific orbitals. Similarly, Spiller et al.^148^ find that when using spin-state-averaged CASSCF orbitals, a subsequent NEVPT2 treatment yields lower magnetic coupling than using spin-pure state-specific orbitals. Angeli and Calzado^157^ suggest using average orbitals of the singlet ground and excited states in the minimal active space to include the ionic contributions and thus ligand–metal delocalization, and Kubas^137^ used spin-averaged Hartree–Fock (SAHF)^170^ orbitals for the low-lying excited-state spectrum of the [FeS] dimer.

On the other hand, CASSCF misses different physical effects, which tends to emphasize the ionic nature of orbitals^136^ and causes MOs of pure ionic wave functions to be too diffuse.^171^ Similarly, Malrieu et al.^155^ showed that the definition of magnetic orbitals from spin-unrestricted density functional theory (DFT) calculations strongly overestimates the metal–ligand delocalization, which might be the reason for the rather large *J* value obtained by BS-DFT^101,147^ and DMRG CASCI calculations based on such orbitals.^100^

#### 4.1.5 CASSCF Effect on Spin–Spin Correlation Function for Fe_2_S_2_

With a spin-adapted basis and the localized and atom-ordered MOs described in the SI, we can use the formulas derived in Appendix B to study the spin–spin interaction between the two magnetic centers in the Fe_2_S_2_ system and the effect of the CASSCF procedure on it.

**Figure 12:**
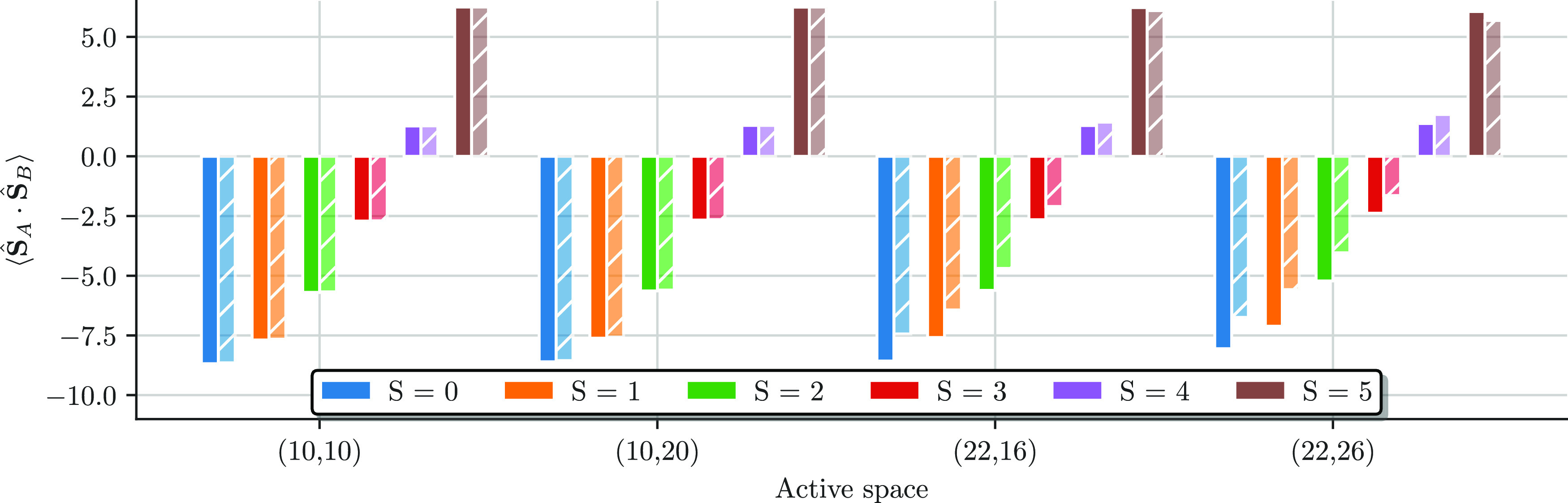
Spin–spin correlation function ⟨**Ŝ**_*A*_ · **Ŝ**_*B*_⟩ between local spins on iron *A* and *B* from CASCI (solid bars) and the final CASSCF results (striped bars) as a function of the active space size for all spin states.

Figure 12 shows the spin–spin correlation function ⟨**Ŝ**_*A*_ · **Ŝ**_*B*_⟩ between the two magnetic centers from the CASCI (solid) and in the CASSCF wave functions (striped bars), as a function of the active space size for all spin states. For all active spaces, the spin–spin alignment changes from antiferromagnetic to ferromagnetic, starting from *S* = 4, as the total spin increases. The spin–spin correlations are somewhat large for the CAS(10e,10o) and CAS(10e,20o), where the CASSCF orbital relaxation does not have a big impact on the expectation values. As for the local spin measurements, the orbital relaxation has the biggest effect in the CAS(22e,26o). The CASSCF procedure has a damping effect on the magnitude of the spin–spin correlations but does not change the description of the underlying physical behavior of a transition from an antiferromagnetic to a ferromagnetic alignment as a function of the total spin.

**Figure 13:**
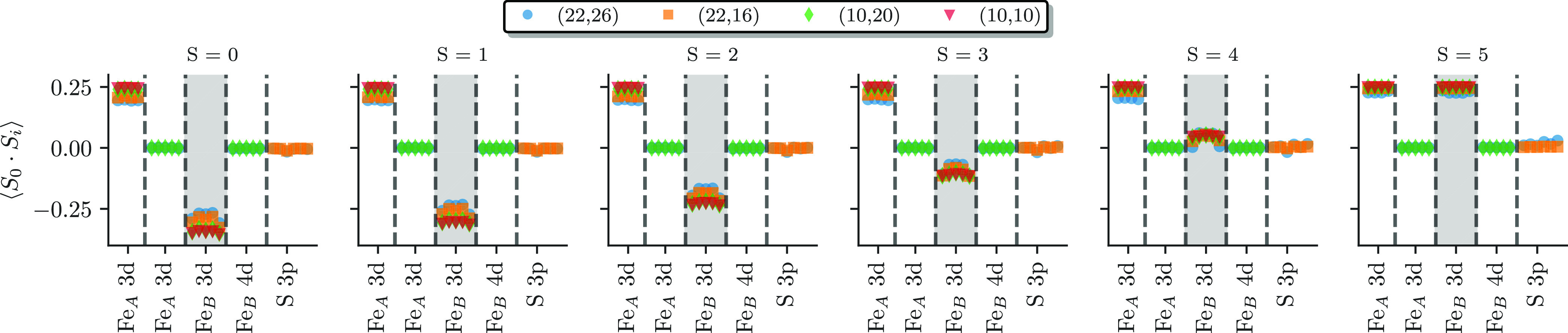
Spin–spin correlation function ⟨**Ŝ**_0_ · **Ŝ**_*i*_⟩ between the first Fe_*A*_ 3d orbital (index 0) and all of the other orbitals *i* obtained via the spin-free RDMs for all of the spin states of the CASSCF results. The x-axis indicates the type of orbitals, where the 3d orbitals of iron *B* are indicated by the gray background. This plot combines all results from the different active spaces indicated by the color and marker types (see the legend and main text).

With access to the 1- and 2-RDMs, we are able to study the spin correlation functions on an orbital-resolved level, including the iron d′ and sulfur 3p orbitals. Figure 13 shows the CASSCF spin–spin correlation function ⟨**Ŝ**_0_ · **Ŝ**_*i*_⟩ between the first Fe_*A*_ 3d orbital and all other orbitals obtained via the spin-free RDMs. Figure 13 contains ⟨**Ŝ**_0_ · **Ŝ**_*i*_⟩ for all spin states, *S* = 0 to *S* = 5 (indicated by the subplot titles), and all active spaces, different colors, and markers. The *x*-axes indicate the different orbitals *i* and different types of orbitals (iron, sulfur, etc.) are separated by vertical dashed lines and data points only show up, when possible, e.g., there are no markers of the (10e,10o) active space results (red triangles) for the iron 4d and sulfur 3p orbitals. The mostly singly occupied first iron A 3d orbital, with index 0, is magnetically parallel aligned to all of the other Fe_*A*_ 3d orbitals, as can be seen by the ⟨**Ŝ**_0_ · **Ŝ**_*i*_⟩ ≈ 0.25, ∀*i* ∈ {Fe_*A*_ 3d}, for all of the spin states. ⟨**Ŝ**_0_ ·**Ŝ**_*i*_⟩ ≈ 1/4 is expected for two ferromagnetically *S* = 1/2 spins. The magnetic 3d orbitals of iron *B* are highlighted by the gray background in Figure 13. Here, one can see that with increasing total spin *S*, indicated by the titles of the subplots, the alignment of the first iron *A* 3d orbital changes from antiferromagnetic, ⟨**Ŝ**_0_ · **Ŝ**_*i*_⟩ < 0, to ferromagnetic alignment, ⟨**Ŝ**_0_ · **Ŝ**_*i*_⟩ ≈ 1/4, ∀*i* ∈ {Fe_*B*_ 3d}, with the 3d orbitals of iron *B*. The results confirm that the exchange interaction exclusively happens between the (magnetic) iron 3d orbitals, while the other orbitals are magnetically inert (indicated by a zero value of ⟨**Ŝ**_0_ · **Ŝ**_*i*_⟩).

### 4.2 Fe_4_S_4_ System

We now turn to the all-ferric [Fe(III)_4_S_4_(SCH_3_)_4_] system. Here, we consider the minimal (20e,20o) active space consisting of the iron 3d orbitals of the four iron atoms. This active space size is already slightly above the current limit of performing routine FCI calculations.^15,16^ Similar to Fe_2_S_2_, we performed state-specific and spin-pure stochastic-CASSCF calculations for all of the spin states, from *S* = 0 up to *S* = 10. We used the geometry studied in refs (63, 100), which is, among other computational details, documented in the SI. We used an ANO-RCC-VDZ basis set for Fe and an ANO-RCC-MB^174^ for all other elements and ensured that the obtained results are converged w.r.t. the number of used walkers *N*_w_. They are already with a very modest *N*_w_ = 1 × 10^6^ walkers.

In the Fe_4_S_4_ study, we use the localized (20e,20o) high-spin ROHF orbitals as a starting guess and focus on the effect of the CASSCF orbital relaxation on the extraction of the model parameters and associated physical and chemical interpretations of the results. Similar as in the FeS dimer case above (section 4.1), we refer to the first iteration of the CASSCF procedure, based on the ROHF orbitals, as CASCI. In our previous work,^63^ we found that the *ab initio* CASCI results can be very well mapped to a simple bilinear Heisenberg model. However, as seen in section 4.1 on the dimer model, orbital relaxation effects can affect the relative energy of the *ab initio* spin states and introduce forms of interactions that go beyond the simple bilinear Heisenberg model.

**Figure 14:**
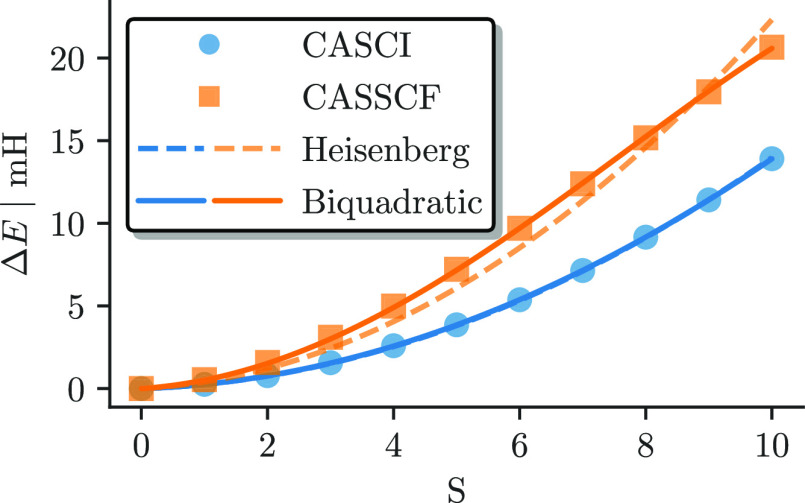
Energy difference to the *S* = 0 ground state (markers) and a simple (dashed) and biquadratic (solid lines) Heisenberg fit for the CASCI (blue) and CASSCF (orange) results of the (20e,20o) active space as a function of the total spin.

Figure 14 shows the energy difference to the *S* = 0 ground state (markers) and a simple (dashed) and a biquadratic (solid line) Heisenberg fit for CASCI and CASSCF results as a function of the total spin. The CASCI results are well represented by a simple Heisenberg model, whereas the CASSCF results differ from it and necessitate a biquadratic model description, similar to the above-studied Fe_2_S_2_ case.

**Figure 15:**
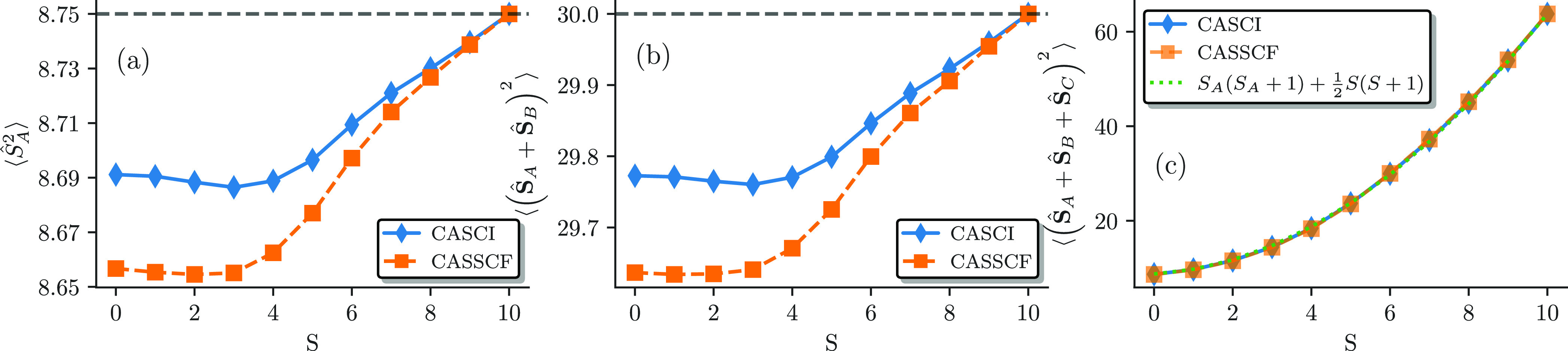
Local spin expectation values for iron *A* (a), *A* + *B* (b), and *A* + *B* + *C* (c) as a function of the total spin for the CASCI (blue) and CASSCF (orange) results.

To investigate the deviation of the *ab initio* CASSCF results from a pure Heisenberg model, we computed the local spin and spin–spin correlation for the Fe_4_S_4_ system. Figure 15 shows the local spin expectation values of iron *A* (a), *A* + *B* (b), and *A* + *B* + *C* (c) as a function of the total spin for the CASCI and CASSCF results. The local spin on the single iron *A* is close to the maximum possible, (*S*_*A*_^max^)^2^ = 8.75, for all of the spin states, and the effect of the CASSCF orbital relaxation is present but small. Due to symmetry reasons, we can safely assume that this expectation value is equal for all four iron centers. The expectation value of the sum of the local spin of the far-distanced irons, *A* and *B*, is close to the maximum possible, (*S*_*AB*_^max^)^2^ = 5(5 + 1) = 30, as can be seen in Figure 15b. This shows that the two far-distanced iron centers are ferromagnetically aligned with *S*_*A*_ + *S*_*B*_ = 5, as already investigated thoroughly for the singlet ground and excited states in ref (63). Again, the orbital relaxation only plays a minor role but shows the same behavior as for the single iron spin. The local spin expectation value of the sum of three irons, ⟨(**Ŝ**_*A*_ + **Ŝ**_*B*_ + **Ŝ**_*C*_)^2^⟩, increases from a minimum value close to (*S*_*ABC*_^min^)^2^ = 8.75 for *S* = 0 all the way to (*S*_*ABC*_^max^)^2^ = ^15^/_2_(^15^/_2_ + 1) = 63.75 for *S* = 10. ⟨(**Ŝ**_*A*_ + **Ŝ**_*B*_ + **Ŝ**_*C*_)^2^⟩ can be represented by *S*_*A*_(*S*_*A*_ + 1) + ^1^/_2_*S*(*S* + 1), as can be seen in the right panel of Figure 15, which is the exact results of a 4-site pure *S* = ^5^/_2_ Heisenberg model. The close agreement to the theoretically maximal values of the local spin is because we investigate the minimal (20e,20o) active space of the magnetic iron 3d orbitals. Inclusion of ligand orbitals would cause a larger deviation similar to the iron dimer studied above and as already anticipated in our previous work.^63^

**Figure 16:**
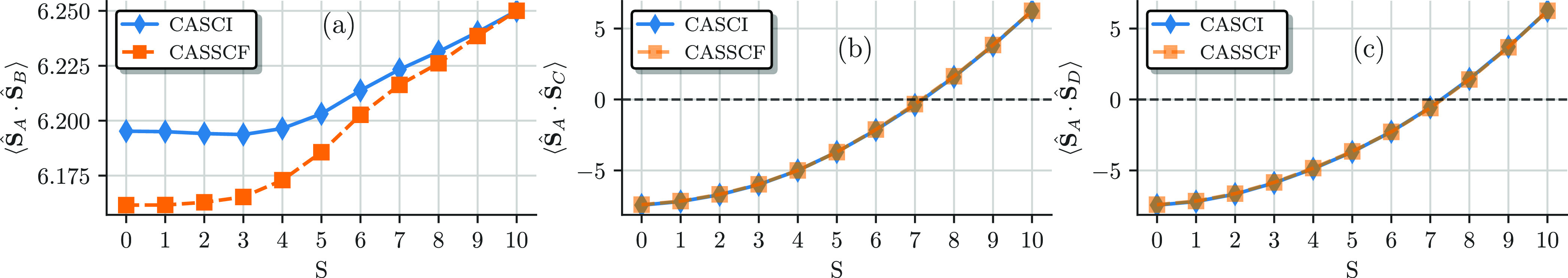
Spin–spin correlation function, ⟨**Ŝ**_*i*_ · **Ŝ**_*j*_⟩, between the four different iron atoms for the CASCI (blue) and CASSCF (orange) results as a function of the total spin. Panel (a) shows the spin–spin interaction between the far-distanced magnetic centers A and B, and panels (b) and (c) show the symmetric interaction between the close-distanced irons, A–C and A–D, respectively.

We now focus on the spin–spin interaction between the four magnetic iron centers. Figure 16 shows the spin–spin correlation function, ⟨**Ŝ**_*i*_ · **Ŝ**_*j*_⟩, between the four different iron atoms for the CASCI and CASSCF results as a function of the total spin. Figure 16a confirms that the two iron atoms with the largest distance, *A* and *B*, always stay ferromagnetically aligned for all of the spin states, with a marginally lowering effect of the CASSCF orbital relaxation. Figure 16b,c shows that the spin–spin interaction between two close-lying iron atoms, e.g., *A* – *C* or *A* – *D*, is antiferromagnetic for the low-spin states and switches to ferromagnetic alignment for *S* = 8 and higher. Additionally, these results confirm that these spin–spin interactions are symmetric and that the CASSCF procedure has only a marginal effect on the obtained expectation values.

## 5 Conclusions

In this work, we present our implementation to compute the spin-pure one- and two-body reduced density matrices, via stochastic sampling, within our spin-adapted FCIQMC implementation. This gives us access to spin-pure two-body observables, such as the spin–spin correlation function, and allows us to use the GUGA-FCIQMC as a spin-pure CI eigensolver in the spin-pure stochastic-CASSCF approach (within OpenMolcas). This, in turn, enables us to stochastically, yet accurately, treat active spaces far larger than conventional CI solvers in a spin-pure manner. The implementation requires only minor modifications to the existing GUGA-FCIQMC implementation and introduces only a small computational overhead. This makes the approach quite efficient and allows us to employ up to hundreds of millions of CSFs simultaneously.

We demonstrate the utility of this method by studying two FeS dimer and tetramer model systems. For the dimer, by performing extensive state-specific CASSCF calculations for the lowest state of each accessible spin-symmetry and four active spaces, we find that (1) the combined effect of Fe 3d orbital relaxation and the ligand-to-metal charge transfer has a larger influence on the energetics of the spin ladder than the sum of the two effects alone. (2) When using (10e,10o) ROHF starting orbitals for the CASSCF procedure, its effect is rather small (few mH) on the singlet–triplet gap, while up to ≈ 20 mH for low-spin–high-spin gap. (3) When one maps *ab initio* results to a (biquadratic) Heisenberg Hamiltonian, performing a spin-pure CASSCF procedure has a large impact on the extracted model parameter.

Access to the spin-pure RDMs with GUGA-FCIQMC allows us to directly measure local spin and spin–spin correlation functions. Insight into these quantities, the local (double) occupation number, and the electron delocalization effect due to the CASSCF procedure enable us to argue why the CASCI results using the (10e,10o) ROHF orbitals agree so well with the bilinear Heisenberg model, while the converged CASSCF do not. The ROHF orbitals are optimized such that the Heisenberg exchange mechanism is the only possible one. Thus, they are too localized on the iron atoms,^155−157^ and even increasing the active space does not enable us to fully capture important spin delocalization and charge fluctuations.^90,100,101^

We study the FeS tetramer in the minimal (20e,20o) active space, which, in a spin-adapted approach, due to 20 open-shell localized 3d orbitals, is a formidable task. Also, for the tetramer, we find that performing a CASSCF procedure necessitates the inclusion of the biquadratic term into the spin model to correctly map the *ab initio* results.

## Figures and Tables

**Figure 17:**
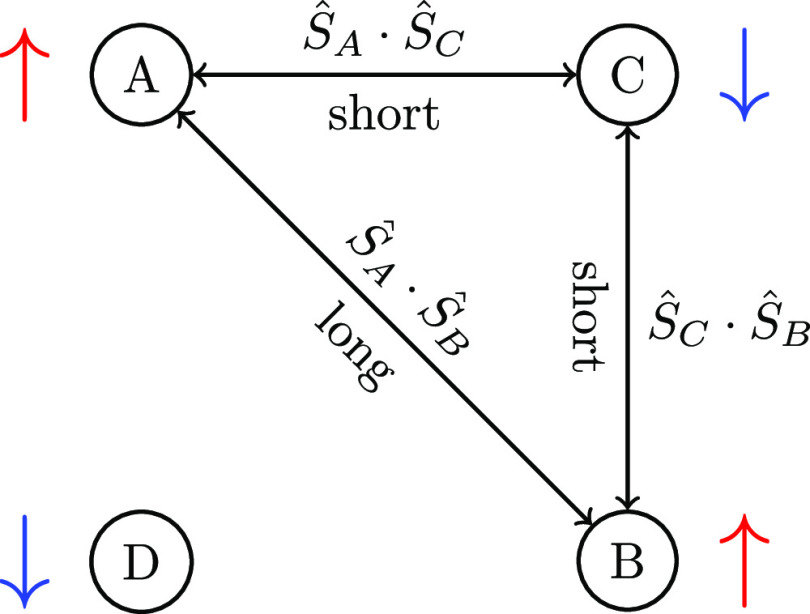
Sketch of the Fe_4_S_4_ geometry.

## Appendix A. Local Spin Measurements

In the GUGA approach, CSFs are spin eigenfunctions up to any spatial orbital *i*. This means that it is straightforward to calculate the expectation value of a cumulative local spin operator **Ŝ_c_**(*i*) consisting of orbitals up to the chosen orbital *i*

20

where **ŝ**_*j*_ indicates the local spin operator of a single molecular orbital (MO) *j*. The square of the operator defined in eq 20, **Ŝ**_c_^2^(*i*), is diagonal in a GUGA-CSF basis, and thus one can straightforwardly calculate the expectation value

21

where *S*_*i*_^μ^ indicated the intermediate total spin of CSF |μ⟩ at the spatial orbital *i*. It is important to note that since eq 21 is a diagonal quantity, the replica method^123^ needs to be used within FCIQMC to obtain unbiased estimates.

Additionally, this necessitates to order orbitals of interest, e.g., to measure the local spin of a specific iron atom, consecutively starting from the beginning, since GUGA CSFs are not spin eigenfunctions for intermediate orbitals. It turns out that this ordering, in conjunction with using localized 3d′ orbitals in the CAS(22e, 26o) for the Fe_2_S_2_ system, is even more optimal as the choice studied in ref (62). More optimal in the sense that we do have an even higher reference weight (0.67 compared to 0.55 for the singlet CASCI), more single reference character, and hence a faster convergence. The detailed orbital choice and ordering used can be found in the SI.

## Appendix B. Spin–Spin Interaction

The measurement of local spin quantities additionally allows us to compute the spin–spin interaction between different iron sites.

### Two Sites

If we assume a set of local, independent spin operators *Ŝ*_*i*_, with [*Ŝ*_*i*_, *Ŝ*_*j*_] = 0 and, due to symmetry, ⟨*Ŝ*_*i*_^2^⟩ = ⟨*Ŝ*_*j*_^2^⟩ ∀*i*, *j*, we can deduce that

from which it follows that

22

Since, following eq 21, we can measure both ⟨(*Ŝ*_*A*_ + *Ŝ*_*B*_)^2^⟩ and ⟨*Ŝ*_*A*_^2^⟩ locally, we can deduce the spin correlation function from purely local spin measurements.

### Three Sites

Next, we consider a model system such as that depicted in Figure 17 with two long bond distances *AB* and *CD* and four remaining short distances and assume ⟨*Ŝ*_*i*_^2^⟩ to be identical for all *i*. If we assume spin correlation functions to be equal for the same bond distances, e.g., ⟨*Ŝ*_*A*_ · *Ŝ*_*B*_⟩ = ⟨*Ŝ*_*C*_ · *Ŝ*_*D*_⟩ and ⟨*Ŝ*_*A*_ · *Ŝ*_*C*_⟩ = ⟨*Ŝ*_*B*_ · *Ŝ*_*C*_⟩, etc. and introduce the notation *Ŝ*_*A*_ + *Ŝ*_*B*_ + *Ŝ*_*C*_ = *Ŝ*_*ABC*_, we can deduce that

from which it follows that

23

Again, we can measure all of the quantities on the right-hand side of eq 23 directly via eq 21.

### Four Sites

Now, we assume: all ⟨*Ŝ*_*i*_^2^⟩ are the same, [*Ŝ*_*i*_, *Ŝ*_*j*_] = 0, ∀ *i*, *j*, ⟨*Ŝ*_*A*_ · *Ŝ*_*B*_⟩ = ⟨*Ŝ*_*C*_ · *Ŝ*_*D*_⟩, ⟨*Ŝ*_*A*_ · *Ŝ*_*C*_⟩ = ⟨*Ŝ*_*B*_ · *Ŝ*_*C*_⟩, and ⟨*Ŝ*_*A*_ · *Ŝ*_*D*_⟩ = ⟨*Ŝ*_*B*_ · *Ŝ*_*D*_⟩. With *Ŝ*_*A*_ + *Ŝ*_*B*_ + *Ŝ*_*C*_ + *Ŝ*_*D*_ = *Ŝ*_*tot*_ for short, we obtain

24

Then plugging eqs 22 and 23 into eq 24 yields

25

with *Ŝ*_*AB*_ = *Ŝ*_*A*_ + *Ŝ*_*B*_ and *Ŝ*_*ABC*_ = *Ŝ*_*A*_ + *Ŝ*_*B*_ + *Ŝ*_*C*_.

## Appendix C. Orbital-Resolved Local Spin and Spin Correlation Function from Spin-Free RDMs

Expressing the local spin operators as^172^

26

with the Pauli matrices^173^

27

and the fermionic annihilation (creation) operators, *a*_*i*,σ_^(†)^, of electrons with spin σ in the spatial orbital *i*. This results in the explicit expressions

28

29

30

where *n*_*i*σ_ = *a*_*i*σ_^†^*a*_*i*σ_ is the fermionic number operator of orbital *i* and spin σ.

If we express the **Ŝ**_*i*_ · **Ŝ**_*j*_ as

31

and consequently, the individual terms as

32

33

34

Combining the *x* and *y* terms yields

35

For *i* = *j*, we can transform eq 35 to

36

For the total local spin operator, this means

37

and consequently, we get the relation

38

With the spin-free excitation operators *Ê*_*ij*_ = ∑_σ_*a*_*i*σ_^†^*a*_*i*σ_ and *ê*_*ij,kl*_ = *Ê*_*ij*_*Ê*_*kl*_ – δ_*jk*_*Ê*_*il*_, we can express eq 38 simply as

39

since *E*_*ii*_ = ∑_σ_*n*_*i*σ_ and *ê*_*ii,ii*_ = *Ê*_*ii*_*Ê*_*ii*_ – *Ê*_*ii*_ = (*n*_*i*↑_ + *n*_*i*↓_)^2^ – *Ê*_*ii*_ = 2*n*_*i*↑_*n*_*i*↓_.

With the spin-free excitation operators, we can write eq 38 as

40

and substituting eq 39 on the lhs of eq 40, we get

41

leading to the relation

42

allowing us to formulate the apparent spin-dependent quantity *S*_*i*_^*z*^2^^ entirely in spin-free terms for *i* = *j*.

For *i* ≠ *j*, we can transform eq 35 to

43

With the observation

44

leading to the relation

45

with which the spin correlation, eq 31, can be expressed as

46

To express eq 46 in spin-free terms, we can rewrite

47

which allows us to write the spin–spin correlation function entirely in spin-free terms as

48

eq 48 allows us to directly obtain the off-diagonal *i* ≠ *j*, spin–spin correlation functions ⟨**Ŝ**_*i*_ · **Ŝ**_*j*_⟩, from the spin-free 2-RDM elements, ⟨*ê*_*ij,ji*_⟩ and ⟨*ê*_*ii,jj*_⟩, on an orbital-resolved level.

## Appendix D. Local Spin and Spin Correlation Functions of a Sum of Orbitals from Spin-Free RDMs

The results from Appendix A and Appendix C can be combined to obtain the local spin, **Ŝ**_c_^2^(*i*) (eq 20), and spin–spin correlation function of a sum of orbitals, e.g., between magnetic centers as in the iron *A* and *B* 3d orbitals, directly from the orbital-resolved spin-free RDMs, ρ_*ij*_ and Γ_*ij,kl*_, respectively, ⟨*Ê*_*ij*_⟩ and ⟨*ê*_*ij,kl*_⟩.

### Local Spin

To obtain the local spin of a set of orbitals , we need to combine eqs 39 and 48 to get

49

### Spin–Spin Correlation Function

Similarly, to obtain the spin–spin correlation function between two sets of orbitals  and , we need to make use of eqs 39 and 48

50

assuming . If the two sets  and  do overlap, eq 50 has to be adapted to use eq 39 in the case *i* = *j*.

The advantage of eqs 49 and 50 compared to the cumulative local spin and the spin correlation functions derived in Appendix B is that they are independent of the ordering of orbitals and do not assume any symmetries of the problem at hand.

## Supporting Information Available

Coordinate (geometry_Fe2S2.xyz, geometry_Fe4S4.xyz), starting (fe2s2_22in26_ROHF.orbitals, fe4s4_ROHF.orbitals) and final OpenMolcas CASSCF orbital files (fe2s2_22in26_*.RasOrb, fe4s4cas20in20_s*.RasOrb) for each spin state of the two studied iron–sulfur systems, as well as sample input files for the FCIQMC (fe2s2_22in26_singlet_fciqmc.input, fe4s4_singlet_fciqmc.input) and OpenMolcas calculations (fe2s2_22in26_singlet_molcas.input, fe4s4_singlet_molcas.input); computational details and comparisons with the available exact results for small active spaces, a table with the data used in Figure 10, a study on improved convergence due to stochastic noise, the protocol of how we compared the orbitals in Figure 11, details on the interface and the RDM storage convention in OpenMolcas, and a quick access literature overview of computational results for the Fe_2_S_2_ system (ZIP)

## References

[ref1] Roos B. O.; Taylor P. R.; Sigbahn P. E. A complete active space SCF method (CASSCF) using a density matrix formulated super-CI approach. Chem. Phys. 1980, 48, 157 10.1016/0301-0104(80)80045-0.

[ref2] Siegbahn P. E. M.; Almlöf J.; Heiberg A.; Roos B. O. The complete active space SCF (CASSCF) method in a Newton–Raphson formulation with application to the HNO molecule. J. Chem. Phys. 1981, 74, 2384 10.1063/1.441359.

[ref3] Helgaker T.; Jørgensen P.; Olsen J. Molecular Electronic-Structure Theory; John Wiley & Sons: Chichester, 2000.

[ref4] Roos B. O. The complete active space SCF method in a fock-matrix-based super-CI formulation. Int. J. Quantum Chem. 1980, 18, 175 10.1002/qua.560180822.

[ref5] Roos B. O. The Complete Active Space Self-Consistent Field Method and its Applications in Electronic Structure Calculations. In Advances in Chemical Physics; John Wiley & Sons, Ltd., 1987; p 399.

[ref6] Li Manni G.; Smart S. D.; Alavi A. Combining the Complete Active Space Self-Consistent Field Method and the Full Configuration Interaction Quantum Monte Carlo within a Super-CI Framework, with Application to Challenging Metal-Porphyrins. J. Chem. Theory Comput. 2016, 12, 1245 10.1021/acs.jctc.5b01190. 26808894

[ref7] Li Manni G.; Guther K.; Ma D.; Dobrautz W. Foundation of Multi-Configurational Quantum Chemistry. In Quantum Chemistry and Dynamics of Excited States; John Wiley & Sons, Ltd., 2020; Chapter 6, p 133.

[ref8] Ruedenberg K.; Sundberg K. R. MCSCF Studies of Chemical Reactions: Natural Reaction Orbitals and Localized Reaction Orbitals. In Quantum Science: Methods and Structure. A Tribute to Per-Olov Löwdin; Calais J.-L.; Goscinski O.; Linderberg J.; Öhrn Y., Eds.; Springer US: Boston, MA, 1976; p 505.

[ref9] Kreplin D. A.; Knowles P. J.; Werner H.-J. Second-order MCSCF optimization revisited. I. Improved algorithms for fast and robust second-order CASSCF convergence. J. Chem. Phys. 2019, 150, 194106 10.1063/1.5094644. 31117783

[ref10] Werner H.; Knowles P. J. A second order multiconfiguration SCF procedure with optimum convergence. J. Chem. Phys. 1985, 82, 5053 10.1063/1.448627.

[ref11] Knowles P. J.; Werner H.-J. An efficient second-order MC SCF method for long configuration expansions. Chem. Phys. Lett. 1985, 115, 259 10.1016/0009-2614(85)80025-7.

[ref12] Lanczos C. An iteration method for the solution of the eigenvalue problem of linear differential and integral operators. J. Res. Natl. Bur. Stand. 1950, 45, 255 10.6028/jres.045.026.

[ref13] Sleijpen G. L. G.; der Vorst H. A. V. A Jacobi–Davidson Iteration Method for Linear Eigenvalue Problems. SIAM Rev. 2000, 42, 267 10.1137/S0036144599363084.

[ref14] Davidson E. R. The iterative calculation of a few of the lowest eigenvalues and corresponding eigenvectors of large real-symmetric matrices. J. Comput. Phys. 1975, 17, 87 10.1016/0021-9991(75)90065-0.

[ref15] Aquilante F.; Autschbach J.; Carlson R. K.; Chibotaru L. F.; Delcey M. G.; De Vico L.; Fdez Galván I.; Ferré N.; Frutos L. M.; Gagliardi L.; Garavelli M.; Giussani A.; Hoyer C. E.; Li Manni G.; Lischka H.; Ma D.; Malmqvist P.-Å.; Müller T.; Nenov A.; Olivucci M.; Pedersen T. B.; Peng D.; Plasser F.; Pritchard B.; Reiher M.; Rivalta I.; Schapiro I.; Segarra-Martí J.; Stenrup M.; Truhlar D. G.; Ungur L.; Valentini A.; Vancoillie S.; Veryazov V.; Vysotskiy V. P.; Weingart O.; Zapata F.; Lindh R. Molcas 8: New capabilities for multiconfigurational quantum chemical calculations across the periodic table. J. Comput. Chem. 2016, 37, 506 10.1002/jcc.24221. 26561362

[ref16] Vacher M.; Alavi A.; Angeli C.; Aquilante F.; Autschbach J.; Bao J. J.; Bokarev S. I.; Bogdanov N. A.; Carlson R. K.; Chibotaru L. F.; Creutzberg J.; Dattani N.; Delcey M. G.; Dong S. S.; Dreuw A.; Freitag L.; Frutos L. M.; Gagliardi L.; Gendron F.; Giussani A.; González L.; Grell G.; Guo M.; Hoyer C. E.; Johansson M.; Keller S.; Knecht S.; Kovačević G.; Källman E.; Li Manni G.; Lundberg M.; Ma Y.; Mai S.; Malhado J. P.; Malmqvist P. Å.; Marquetand P.; Mewes S. A.; Norell J.; Olivucci M.; Oppel M.; Phung Q. M.; Pierloot K.; Plasser F.; Reiher M.; Sand A. M.; Schapiro I.; Sharma P.; Stein C. J.; Sørensen L. K.; Truhlar D. G.; Ugandi M.; Ungur L.; Valentini A.; Vancoillie S.; Veryazov V.; Weser O.; Wesołowski T. A.; Widmark P.-O.; Wouters S.; Zech A.; Zobel J. P.; Lindh R.; et al. OpenMolcas: From Source Code to Insight. J. Chem. Theory Comput. 2019, 15, 5925 10.1021/acs.jctc.9b00532. 31509407

[ref17] Vogiatzis K. D.; Ma D.; Olsen J.; Gagliardi L.; de Jong W. A. Pushing configuration-interaction to the limit: Towards massively parallel MCSCF calculations. J. Chem. Phys. 2017, 147, 184111 10.1063/1.4989858. 29141437

[ref18] White S. R. Density matrix formulation for quantum renormalization groups. Phys. Rev. Lett. 1992, 69, 2863 10.1103/PhysRevLett.69.2863. 10046608

[ref19] White S. R. Density-matrix algorithms for quantum renormalization groups. Phys. Rev. B 1993, 48, 10345 10.1103/PhysRevB.48.10345. 10007313

[ref20] Schollwöck U. The density-matrix renormalization group in the age of matrix product states. Ann. Phys. 2011, 326, 96 10.1016/j.aop.2010.09.012.

[ref21] Schollwöck U. The density-matrix renormalization group. Rev. Mod. Phys. 2005, 77, 259 10.1103/RevModPhys.77.259.

[ref22] Nakatani N.; Guo S. Density matrix renormalization group (DMRG) method as a common tool for large active-space CASSCF/CASPT2 calculations. J. Chem. Phys. 2017, 146, 094102 10.1063/1.4976644.

[ref23] Zgid D.; Nooijen M. The density matrix renormalization group self-consistent field method: Orbital optimization with the density matrix renormalization group method in the active space. J. Chem. Phys. 2008, 128, 144116 10.1063/1.2883981. 18412432

[ref24] Ghosh D.; Hachmann J.; Yanai T.; Chan G. K.-L. Orbital optimization in the density matrix renormalization group, with applications to polyenes and β-carotene. J. Chem. Phys. 2008, 128, 144117 10.1063/1.2883976. 18412433

[ref25] Brabec J.; Brandejs J.; Kowalski K.; Xantheas S.; Legeza O.; Veis L. Massively parallel quantum chemical density matrix renormalization group method. J. Comput. Chem. 2021, 42, 534 10.1002/jcc.26476. 33377527

[ref26] Chan G. K.-L.; Sharma S. The Density Matrix Renormalization Group in Quantum Chemistry. Annu. Rev. Phys. Chem. 2011, 62, 465 10.1146/annurev-physchem-032210-103338. 21219144

[ref27] Sharma S.; Chan G. K.-L. Spin-adapted density matrix renormalization group algorithms for quantum chemistry. J. Chem. Phys. 2012, 136, 124121 10.1063/1.3695642. 22462849

[ref28] Marti K. H.; Reiher M. The Density Matrix Renormalization Group Algorithm in Quantum Chemistry. Z. Phys. Chem. 2010, 224, 583 10.1524/zpch.2010.6125.

[ref29] Keller S.; Dolfi M.; Troyer M.; Reiher M. An efficient matrix product operator representation of the quantum chemical Hamiltonian. J. Chem. Phys. 2015, 143, 244118 10.1063/1.4939000. 26723662

[ref30] Ma Y.; Knecht S.; Keller S.; Reiher M. Second-Order Self-Consistent-Field Density-Matrix Renormalization Group. J. Chem. Theory Comput. 2017, 13, 2533 10.1021/acs.jctc.6b01118. 28485978

[ref31] Guther K.; Anderson R. J.; Blunt N. S.; Bogdanov N. A.; Cleland D.; Dattani N.; Dobrautz W.; Ghanem K.; Jeszenszki P.; Liebermann N.; Li Manni G.; Lozovoi A. Y.; Luo H.; Ma D.; Merz F.; Overy C.; Rampp M.; Samanta P. K.; Schwarz L. R.; Shepherd J. J.; Smart S. D.; Vitale E.; Weser O.; Booth G. H.; Alavi A. NECI: N-Electron Configuration Interaction with an emphasis on state-of-the-art stochastic methods. J. Chem. Phys. 2020, 153, 034107 10.1063/5.0005754. 32716189

[ref32] Booth G. H.; Smart S. D.; Alavi A. Linear-scaling and parallelisable algorithms for stochastic quantum chemistry. Mol. Phys. 2014, 112, 1855 10.1080/00268976.2013.877165.

[ref33] Booth G. H.; Thom A. J. W.; Alavi A. Fermion Monte Carlo without fixed nodes: A game of life, death, and annihilation in Slater determinant space. J. Chem. Phys. 2009, 131, 054106 10.1063/1.3193710. 19673550

[ref34] Cleland D.; Booth G. H.; Alavi A. Communications: Survival of the fittest: Accelerating convergence in full configuration-interaction quantum Monte Carlo. J. Chem. Phys. 2010, 132, 041103 10.1063/1.3302277. 20113011

[ref35] Thomas R. E.; Sun Q.; Alavi A.; Booth G. H. Stochastic Multiconfigurational Self-Consistent Field Theory. J. Chem. Theory Comput. 2015, 11, 5316 10.1021/acs.jctc.5b00917. 26894240

[ref36] Huron B.; Malrieu J. P.; Rancurel P. Iterative perturbation calculations of ground and excited state energies from multiconfigurational zeroth-order wavefunctions. J. Chem. Phys. 1973, 58, 5745 10.1063/1.1679199.

[ref37] Evangelisti S.; Daudey J.-P.; Malrieu J.-P. Convergence of an improved CIPSI algorithm. Chem. Phys. 1983, 75, 91 10.1016/0301-0104(83)85011-3.

[ref38] Garniron Y.; Scemama A.; Loos P.-F.; Caffarel M. Hybrid stochastic-deterministic calculation of the second-order perturbative contribution of multireference perturbation theory. J. Chem. Phys. 2017, 147, 034101 10.1063/1.4992127. 28734281

[ref39] Levine D. S.; Hait D.; Tubman N. M.; Lehtola S.; Whaley K. B.; Head-Gordon M. CASSCF with Extremely Large Active Spaces Using the Adaptive Sampling Configuration Interaction Method. J. Chem. Theory Comput. 2020, 16, 2340 10.1021/acs.jctc.9b01255. 32109055

[ref40] Smith J. E. T.; Mussard B.; Holmes A. A.; Sharma S. Cheap and Near Exact CASSCF with Large Active Spaces. J. Chem. Theory Comput. 2017, 13, 5468 10.1021/acs.jctc.7b00900. 28968097

[ref41] Sharma S.; Holmes A. A.; Jeanmairet G.; Alavi A.; Umrigar C. J. Semistochastic Heat-Bath Configuration Interaction Method: Selected Configuration Interaction with Semistochastic Perturbation Theory. J. Chem. Theory Comput. 2017, 13, 1595 10.1021/acs.jctc.6b01028. 28263594

[ref42] Holmes A. A.; Tubman N. M.; Umrigar C. J. Heat-Bath Configuration Interaction: An Efficient Selected Configuration Interaction Algorithm Inspired by Heat-Bath Sampling. J. Chem. Theory Comput. 2016, 12, 3674 10.1021/acs.jctc.6b00407. 27428771

[ref43] Tubman N. M.; Freeman C. D.; Levine D. S.; Hait D.; Head-Gordon M.; Whaley K. B. Modern Approaches to Exact Diagonalization and Selected Configuration Interaction with the Adaptive Sampling CI Method. J. Chem. Theory Comput. 2020, 16, 2139 10.1021/acs.jctc.8b00536. 32159951

[ref44] Tubman N. M.; Lee J.; Takeshita T. Y.; Head-Gordon M.; Whaley K. B. A deterministic alternative to the full configuration interaction quantum Monte Carlo method. J. Chem. Phys. 2016, 145, 044112 10.1063/1.4955109. 27475353

[ref45] Garniron Y.; Scemama A.; Giner E.; Caffarel M.; Loos P.-F. Selected configuration interaction dressed by perturbation. J. Chem. Phys. 2018, 149, 064103 10.1063/1.5044503. 30111155

[ref46] Zhang N.; Liu W.; Hoffmann M. R. Iterative Configuration Interaction with Selection. J. Chem. Theory Comput. 2020, 16, 2296 10.1021/acs.jctc.9b01200. 32069046

[ref47] Chilkuri V. G.; Neese F. Comparison of many-particle representations for selected-CI I: A tree based approach. J. Comput. Chem. 2021, 42, 982 10.1002/jcc.26518. 33764585

[ref48] Chilkuri V. G.; Neese F. Comparison of Many-Particle Representations for Selected Configuration Interaction: II. Numerical Benchmark Calculations. J. Chem. Theory Comput. 2021, 17, 2868 10.1021/acs.jctc.1c00081. 33886300 PMC8279407

[ref49] Bytautas L.; Ruedenberg K. Correlation energy extrapolation by intrinsic scaling. I. Method and application to the neon atom. J. Chem. Phys. 2004, 121, 10905 10.1063/1.1811603. 15634041

[ref50] Bytautas L.; Ruedenberg K. Correlation energy extrapolation by intrinsic scaling. II. The water and the nitrogen molecule. J. Chem. Phys. 2004, 121, 10919 10.1063/1.1811604. 15634042

[ref51] Ivanic J. Direct configuration interaction and multiconfigurational self-consistent-field method for multiple active spaces with variable occupations. I. Method. J. Chem. Phys. 2003, 119, 9364–9376. 10.1063/1.1615954.

[ref52] Ivanic J. Direct configuration interaction and multiconfigurational self-consistent-field method for multiple active spaces with variable occupations. II. Application to oxoMn(salen) and N_2_O_4_. J. Chem. Phys. 2003, 119, 9377–9385. 10.1063/1.1615955.

[ref53] Ma D.; Li Manni G.; Gagliardi L. The generalized active space concept in multiconfigurational self-consistent field methods. J. Chem. Phys. 2011, 135, 044128 10.1063/1.3611401. 21806111

[ref54] Vogiatzis K. D.; Li Manni G.; Stoneburner S. J.; Ma D.; Gagliardi L. Systematic Expansion of Active Spaces beyond the CASSCF Limit: A GASSCF/SplitGAS Benchmark Study. J. Chem. Theory Comput. 2015, 11, 3010 10.1021/acs.jctc.5b00191. 26575738

[ref55] Weser O.; Freitag L.; Guther K.; Alavi A.; Li Manni G. Chemical insights into the electronic structure of Fe(II) porphyrin using FCIQMC, DMRG, and generalized active spaces. Int. J. Quantum Chem. 2021, 121, e26454 10.1002/qua.26454.

[ref56] Krylov A. I. Spin-flip configuration interaction: an electronic structure model that is both variational and size-consistent. Chem. Phys. Lett. 2001, 350, 522–530. 10.1016/S0009-2614(01)01316-1.

[ref57] Mato J.; Gordon M. S. A general spin-complete spin-flip configuration interaction method. Phys. Chem. Chem. Phys. 2018, 20, 2615–2626. 10.1039/C7CP06837A. 29319079

[ref58] Li Manni G.; Alavi A. Understanding the Mechanism Stabilizing Intermediate Spin States in Fe(II)-Porphyrin. J. Phys. Chem. A 2018, 122, 4935 10.1021/acs.jpca.7b12710. 29595978

[ref59] Bogdanov N. A.; Li Manni G.; Sharma S.; Gunnarsson O.; Alavi A. New superexchange paths due to breathing-enhanced hopping in corner-sharing cuprates. arXiv:1803.07026 2018.

[ref60] Li Manni G.; Kats D.; Tew D. P.; Alavi A. Role of Valence and Semicore Electron Correlation on Spin Gaps in Fe(II)-Porphyrins. J. Chem. Theory Comput. 2019, 15, 1492 10.1021/acs.jctc.8b01277. 30681852 PMC6728061

[ref61] Sun Q.; Yang J.; Chan G. K.-L. A general second order complete active space self-consistent-field solver for large-scale systems. Chem. Phys. Lett. 2017, 683, 291 10.1016/j.cplett.2017.03.004.

[ref62] Li Manni G.; Dobrautz W.; Alavi A. Compression of Spin-Adapted Multiconfigurational Wave Functions in Exchange-Coupled Polynuclear Spin Systems. J. Chem. Theory Comput. 2020, 16, 2202 10.1021/acs.jctc.9b01013. 32053374 PMC7307909

[ref63] Li Manni G.; Dobrautz W.; Bogdanov N. A.; Guther K.; Alavi A. Resolution of Low-Energy States in Spin-Exchange Transition-Metal Clusters: Case Study of Singlet States in [Fe(III)_4_S_4_] Cubanes. J. Phys. Chem. A 2021, 125, 22 10.1021/acs.jpca.1c00397. PMC8201447 34048648

[ref64] Li Manni G. Modeling Magnetic Interactions in High-Valent Trinuclear [Mn(IV)_3_O_4_]^4+^ Complexes Through Highly Compressed Multi-Configurational Wave Functions. Phys. Chem. Chem. Phys. 2021, 10.1039/D1CP03259C. 34525156

[ref65] Dobrautz W.; Smart S. D.; Alavi A. Efficient formulation of full configuration interaction quantum Monte Carlo in a spin eigenbasis via the graphical unitary group approach. J. Chem. Phys. 2019, 151, 094104 10.1063/1.5108908. 31492066

[ref66] Booth G. H.; Alavi A. Standalone NECI codebase designed for FCIQMC and other stochastic quantum chemistry methods. https://github.com/ghb24/NECI_STABLE, 2013.

[ref67] Beinert H.; Holm R. H.; Münck E. Iron-Sulfur Clusters: Nature’s Modular, Multipurpose Structures. Science 1997, 277, 653–659. 10.1126/science.277.5326.653. 9235882

[ref68] ÖSTERBERG R. Origins of metal ions in biology. Nature 1974, 249, 382 10.1038/249382a0. 4842310

[ref69] Howard J. B.; Rees D. C. Structural Basis of Biological Nitrogen Fixation. Chem. Rev. 1996, 96, 2965 10.1021/cr9500545. 11848848

[ref70] Vollmer S. J.; Switzer R. L.; Debrunner P. G. Oxidation-reduction properties of the iron-sulfur cluster in Bacillus subtilis glutamine phosphoribosylpyrophosphate amidotransferase. J. Biol. Chem. 1983, 258, 14284 10.1016/S0021-9258(17)43858-0. 6315725

[ref71] Stombaugh N. A.; Sundquist J. E.; Burris R. H.; Orme-Johnson W. H. Oxidation-reduction properties of several low potential iron-sulfur proteins and of methylviologen. Biochemistry 1976, 15, 2633 10.1021/bi00657a024. 181047

[ref72] Rees D. C.; Howard J. B. The Interface Between the Biological and Inorganic Worlds: Iron-Sulfur Metalloclusters. Science 2003, 300, 929 10.1126/science.1083075. 12738849

[ref73] Hudson J. M.; Heffron K.; Kotlyar V.; Sher Y.; Maklashina E.; Cecchini G.; Armstrong F. A. Electron Transfer and Catalytic Control by the Iron-Sulfur Clusters in a Respiratory Enzyme, *E.coli* Fumarate Reductase. J. Am. Chem. Soc. 2005, 127, 6977 10.1021/ja043404q. 15884941

[ref74] Mortenson L.; Valentine R.; Carnahan J. An electron transport factor from Clostridiumpasteurianum. Biochem. Biophys. Res. Commun. 1962, 7, 448 10.1016/0006-291X(62)90333-9. 14476372

[ref75] Tagawa K.; Arnon D. I. Ferredoxins as Electron Carriers in Photosynthesis and in the Biological Production and Consumption of Hydrogen Gas. Nature 1962, 195, 537 10.1038/195537a0. 14039612

[ref76] Lubitz W.; Ogata H.; Rüdiger O.; Reijerse E. Hydrogenases. Chem. Rev. 2014, 114, 4081 10.1021/cr4005814. 24655035

[ref77] Blondin G.; Girerd J. J. Interplay of electron exchange and electron transfer in metal polynuclear complexes in proteins or chemical models. Chem. Rev. 1990, 90, 1359 10.1021/cr00106a001.

[ref78] Han A.-L.; Yagi T.; Hatefi Y. Studies on the structure of NADH:ubiquinone oxidoreductase complex: Topography of the subunits of the iron-sulfur protein component. Arch. Biochem. Biophys. 1989, 275, 166 10.1016/0003-9861(89)90360-3. 2510601

[ref79] Mitchell P. The Correlation of Chemical and Osmotic Forces in Biochemistry. J. Biochem. 1985, 97, 1 10.1093/oxfordjournals.jbchem.a135033. 2581936

[ref80] Golbeck J. H. Structure, function and organization of the photosystem I reaction center complex. Biochim. Biophys. Acta, Bioenerg. 1987, 895, 167 10.1016/S0304-4173(87)80002-2. 3333014

[ref81] Peters J. W.; Stowell M. H. B.; Soltis S. M.; Finnegan M. G.; Johnson M. K.; Rees D. C. Redox-Dependent Structural Changes in the Nitrogenase P-Cluster. Biochemistry 1997, 36, 1181 10.1021/bi9626665. 9063865

[ref82] Ibrahim I. M.; Wu H.; Ezhov R.; Kayanja G. E.; Zakharov S. D.; Du Y.; Tao W. A.; Pushkar Y.; Cramer W. A.; Puthiyaveetil S. An evolutionarily conserved iron-sulfur cluster underlies redox sensory function of the Chloroplast Sensor Kinase. Commun. Biol. 2020, 3, 13 10.1038/s42003-019-0728-4. 31925322 PMC6949291

[ref83] Johnson D. C.; Dean D. R.; Smith A. D.; Johnson M. K. Structure, function and formation of biological iron-sulfur clusters. Annu. Rev. Biochem. 2005, 74, 247 10.1146/annurev.biochem.74.082803.133518. 15952888

[ref84] Noodleman L.; Peng C.; Case D.; Mouesca J.-M. Orbital interactions, electron delocalization and spin coupling in iron-sulfur clusters. Coord. Chem. Rev. 1995, 144, 199 10.1016/0010-8545(95)07011-L.

[ref85] Van Kuiken B. E.; Hahn A. W.; Nayyar B.; Schiewer C. E.; Lee S. C.; Meyer F.; Weyhermüller T.; Nicolaou A.; Cui Y.-T.; Miyawaki J.; Harada Y.; DeBeer S. Electronic Spectra of Iron–Sulfur Complexes Measured by 2p3d RIXS Spectroscopy. Inorg. Chem. 2018, 57, 7355 10.1021/acs.inorgchem.8b01010. 29847108

[ref86] Eaton W. A.; Palmer G.; Fee J. A.; Kimura T.; Lovenberg W. Tetrahedral Iron in the Active Center of Plant Ferredoxins and Beef Adrenodoxin. Proc. Natl. Acad. Sci. U.S.A. 1971, 68, 3015 10.1073/pnas.68.12.3015. 4332004 PMC389581

[ref87] Heisenberg W. Zur Theorie des Ferromagnetismus. Z. Phys. 1928, 49, 619 10.1007/BF01328601.

[ref88] Dirac P. A. M. On the theory of quantum mechanics. Proc. R. Soc. London, Ser. A 1926, 112, 661 10.1098/rspa.1926.0133.

[ref89] Boča R. Chapter 10 - Dinuclear Systems. In Theoretical Foundations of Molecular Magnetism; Boča R., Ed.; Current Methods in Inorganic Chemistry; Elsevier, 1999; Vol. 1, p 579.

[ref90] Labèguerie P.; Boilleau C.; Bastardis R.; Suaud N.; Guihéry N.; Malrieu J.-P. Is it possible to determine rigorous magnetic Hamiltonians in spin s=1 systems from density functional theory calculations?. J. Chem. Phys. 2008, 129, 154110 10.1063/1.2993263. 19045179

[ref91] Kittel C. Model of Exchange-Inversion Magnetization. Phys. Rev. 1960, 120, 335 10.1103/PhysRev.120.335.

[ref92] Anderson P. W. New Approach to the Theory of Superexchange Interactions. Phys. Rev. 1959, 115, 2 10.1103/PhysRev.115.2.

[ref93] Falk U.; Furrer A.; Kjems J. K.; Güdel H. U. Biquadratic Exchange in CsMn_*x*_Mg_1–*x*_Br_3_. Phys. Rev. Lett. 1984, 52, 1336 10.1103/PhysRevLett.52.1336.

[ref94] Bastardis R.; Guihéry N.; de Graaf C. Isotropic non-Heisenberg terms in the magnetic coupling of transition metal complexes. J. Chem. Phys. 2008, 129, 104102 10.1063/1.2975336. 19044903

[ref95] Calzado C. J.; Malrieu J.-P.; Sanz J. F. Physical Factors Governing the Amplitude of the Electron Transfer Integral in Mixed-Valence Compounds. J. Phys. Chem. A 1998, 102, 3659 10.1021/jp980105c.

[ref96] Bastardis R.; Guihéry N.; de Graaf C. Microscopic origin of isotropic non-Heisenberg behavior in *S* = 1 magnetic systems. Phys. Rev. B 2007, 76, 132412 10.1103/PhysRevB.76.132412.

[ref97] Moreira I. d. P. R.; Suaud N.; Guihéry N.; Malrieu J. P.; Caballol R.; Bofill J. M.; Illas F. Derivation of spin Hamiltonians from the exact Hamiltonian: Application to systems with two unpaired electrons per magnetic site. Phys. Rev. B 2002, 66, 134430 10.1103/PhysRevB.66.134430.

[ref98] Gillum W. O.; Frankel R. B.; Foner S.; Holm R. H. Synthetic analogues of the active sites of iron-sulfur proteins. XIII. Further electronic structural relationships between the analogues [Fe_2_S_2_(SR)_4_]^2–^ and the active sites of oxidized 2Fe-2S proteins. Inorg. Chem. 1976, 15, 1095 10.1021/ic50159a023.

[ref99] Palmer G.; Dunham W.; Fee J.; Sands R.; Iizuka T.; Yonetani T. The magnetic susceptibility of spinach ferredoxin from 77–250°K: A measurement of the antiferromagnetic coupling between the two iron atoms. Biochim. Biophys. Acta, Bioenerg. 1971, 245, 201 10.1016/0005-2728(71)90022-3. 5132472

[ref100] Sharma S.; Sivalingam K.; Neese F.; Chan G. K.-L. Low-energy spectrum of iron–sulfur clusters directly from many-particle quantum mechanics. Nat. Chem. 2014, 6, 927 10.1038/nchem.2041. 25242489

[ref101] Noodleman L.; Case D. A. Density-Functional Theory of Spin Polarization and Spin Coupling in Iron—Sulfur Clusters. In Advances in Inorganic Chemistry; Cammack R., Ed.; Academic Press, 1992; Vol. 38, p 423.

[ref103] Dobrautz W. Development of Full Configuration Interaction Quantum Monte Carlo Methods for Strongly Correlated Electron Systems. Ph.D. thesis, University of Stuttgart, 2019.

[ref104] Paldus J. Group theoretical approach to the configuration interaction and perturbation theory calculations for atomic and molecular systems. J. Chem. Phys. 1974, 61, 5321 10.1063/1.1681883.

[ref105] Paldus J. Matrix elements of unitary group generators in many-fermion correlation problem. II. Graphical methods of spin algebras. J. Math. Chem. 2021, 59, 37 10.1007/s10910-020-01173-8.

[ref106] Shavitt I. Graph theoretical concepts for the unitary group approach to the many-electron correlation problem. Int. J. Quantum Chem. 1977, 12, 131 10.1002/qua.560120819.

[ref107] Shavitt I. Matrix element evaluation in the unitary group approach to the electron correlation problem. Int. J. Quantum Chem. 1978, 14, 5–32. 10.1002/qua.560140803.

[ref108] Matsen F. Spin-Free Quantum Chemistry. In Advances in Quantum Chemistry; Elsevier, 1964; p 59.

[ref109] Paldus J. A pattern calculus for the unitary group approach to the electronic correlation problem. Int. J. Quantum Chem. 1975, 9, 165 10.1002/qua.560090823.

[ref110] Paldus J. Unitary-group approach to the many-electron correlation problem: Relation of Gelfand and Weyl tableau formulations. Phys. Rev. A 1976, 14, 1620 10.1103/PhysRevA.14.1620.

[ref111] Gel’fand I. M.; Cetlin M. L. Finite-dimensional representations of the group of unimodular matrices. Dokl. Akad. Nauk SSSR 1950, 71, 825.

[ref112] Gel’fand I. M.; Cetlin M. L. Finite-dimensional representations of the group of orthogonal matrices. Dokl. Akad. Nauk SSSR 1950, 71, 1017.

[ref113] Gel’fand I. M. The center of an infinitesimal group ring. Mat. Sb. (N.S.) 1950, 26, 103.

[ref114] Shavitt I. The Graphical Unitary Group Approach and Its Application to Direct Configuration Interaction Calculations. In The Unitary Group for the Evaluation of Electronic Energy Matrix Elements; Hinze J., Ed.; Springer Berlin Heidelberg: Berlin, Heidelberg, 1981; p 51.

[ref115] Brooks B. R.; Schaefer H. F. The graphical unitary group approach to the electron correlation problem. Methods and preliminary applications. J. Chem. Phys. 1979, 70, 5092 10.1063/1.437351.

[ref116] Brooks B. R.; Laidig W. D.; Saxe P.; Handy N. C. III; H F S The Loop-Driven Graphical Unitary Group Approach: A Powerful Method for the Variational Description of Electron Correlation. Phys. Scr. 1980, 21, 312 10.1088/0031-8949/21/3-4/013.

[ref117] Barca G. M. J.; Bertoni C.; Carrington L.; Datta D.; De Silva N.; Deustua J. E.; Fedorov D. G.; Gour J. R.; Gunina A. O.; Guidez E.; Harville T.; Irle S.; Ivanic J.; Kowalski K.; Leang S. S.; Li H.; Li W.; Lutz J. J.; Magoulas I.; Mato J.; Mironov V.; Nakata H.; Pham B. Q.; Piecuch P.; Poole D.; Pruitt S. R.; Rendell A. P.; Roskop L. B.; Ruedenberg K.; Sattasathuchana T.; Schmidt M. W.; Shen J.; Slipchenko L.; Sosonkina M.; Sundriyal V.; Tiwari A.; Galvez Vallejo J. L.; Westheimer B.; Wloch M.; Xu P.; Zahariev F.; Gordon M. S. Recent developments in the general atomic and molecular electronic structure system. J. Chem. Phys. 2020, 152, 154102 10.1063/5.0005188. 32321259

[ref118] Andersson K.; Malmqvist P.-Å.; Roos B. O. Second-order perturbation theory with a complete active space self-consistent field reference function. J. Chem. Phys. 1992, 96, 1218 10.1063/1.462209.

[ref119] Ma D.; Li Manni G.; Olsen J.; Gagliardi L. Second-Order Perturbation Theory for Generalized Active Space Self-Consistent-Field Wave Functions. J. Chem. Theory Comput. 2016, 12, 3208 10.1021/acs.jctc.6b00382. 27276688

[ref120] Li Manni G.; Ma D.; Aquilante F.; Olsen J.; Gagliardi L. SplitGAS Method for Strong Correlation and the Challenging Case of Cr2. J. Chem. Theory Comput. 2013, 9, 3375 10.1021/ct400046n. 26584093

[ref121] Blunt N. S.; Smart S. D.; Booth G. H.; Alavi A. An excited-state approach within full configuration interaction quantum Monte Carlo. J. Chem. Phys. 2015, 143, 134117 10.1063/1.4932595. 26450302

[ref122] Blunt N. S.; Booth G. H.; Alavi A. Density matrices in full configuration interaction quantum Monte Carlo: Excited states, transition dipole moments, and parallel distribution. J. Chem. Phys. 2017, 146, 244105 10.1063/1.4986963. 28668027

[ref123] Overy C.; Booth G. H.; Blunt N. S.; Shepherd J. J.; Cleland D.; Alavi A. Unbiased reduced density matrices and electronic properties from full configuration interaction quantum Monte Carlo. J. Chem. Phys. 2014, 141, 244117 10.1063/1.4904313. 25554143

[ref124] Holmes A. A.; Changlani H. J.; Umrigar C. J. Efficient Heat-Bath Sampling in Fock Space. J. Chem. Theory Comput. 2016, 12, 1561 10.1021/acs.jctc.5b01170. 26959242

[ref125] Neufeld V. A.; Thom A. J. W. Exciting Determinants in Quantum Monte Carlo: Loading the Dice with Fast, Low-Memory Weights. J. Chem. Theory Comput. 2019, 15, 127 10.1021/acs.jctc.8b00844. 30359533

[ref126] Smart S.; Booth G.; Alavi A. unpublished.

[ref127] Smart S. D. The use of spin-pure and non-orthogonal Hilbert spaces in Full Configuration Interaction Quantum Monte-Carlo. Ph.D. thesis, University of Cambridge, 2013.

[ref128] Luzanov A. V. Calculating spin density in the quantum-chemical unitary formalism. Theor. Exp. Chem. 1985, 21, 329 10.1007/BF00523996.

[ref129] Scemama A.; Giner E. An efficient implementation of Slater-Condon rules. arXiv preprint arXiv:1311.6244, 2013.

[ref130] Blunt N. S.; Smart S. D.; Kersten J. A. F.; Spencer J. S.; Booth G. H.; Alavi A. Semi-stochastic full configuration interaction quantum Monte Carlo: Developments and application. J. Chem. Phys. 2015, 142, 184107 10.1063/1.4920975. 25978883

[ref131] Petruzielo F. R.; Holmes A. A.; Changlani H. J.; Nightingale M. P.; Umrigar C. J. Semistochastic Projector Monte Carlo Method. Phys. Rev. Lett. 2012, 109, 230201 10.1103/PhysRevLett.109.230201. 23368167

[ref132] Mayerle J. J.; Frankel R. B.; Holm R. H.; Ibers J. A.; Phillips W. D.; Weiher J. F. Synthetic Analogs of the Active Sites of Iron-Sulfur Proteins. Structure and Properties of Bis[o-xylyldithiolato-μ_2_-sulfidoferrate(III)], an Analog of the 2Fe-2S Proteins. Proc. Natl. Acad. Sci. U.S.A. 1973, 70, 2429 10.1073/pnas.70.8.2429. 4525429 PMC433750

[ref133] Mayerle J. J.; Denmark S. E.; DePamphilis B. V.; Ibers J. A.; Holm R. H. Synthetic analogs of the active sites of iron-sulfur proteins. XI. Synthesis and properties of complexes containing the iron sulfide (Fe2S2) core and the structures of bis[o-xylyl-α, α’-dithiolato-μ-sulfido-ferrate(III)] and bis[p-tolylthiolato-μ-sulfido-ferrate(III)] dianions. J. Am. Chem. Soc. 1975, 97, 1032–1045. 10.1021/ja00838a015.

[ref134] Averill B. A.; Herskovitz T.; Holm R. H.; Ibers J. A. Synthetic analogs of the active sites of iron-sulfur proteins. II. Synthesis and structure of the tetra[mercapto-μ_3_-sulfido-iron] clusters, [Fe_4_S_4_(SR)_4_]^2–^. J. Am. Chem. Soc. 1973, 95, 3523 10.1021/ja00792a013. 4708377

[ref135] Cho D.; Rouxel J. R.; Mukamel S.; Chan G. K.-L.; Li Z. Stimulated X-ray Raman and Absorption Spectroscopy of Iron–Sulfur Dimers. J. Phys. Chem. Lett. 2019, 10, 6664 10.1021/acs.jpclett.9b02414. 31532691

[ref136] Chilkuri V. G.; DeBeer S.; Neese F. Ligand Field Theory and Angular Overlap Model Based Analysis of the Electronic Structure of Homovalent Iron–Sulfur Dimers. Inorg. Chem. 2020, 59, 984 10.1021/acs.inorgchem.9b00974. 31247844 PMC6978809

[ref137] Kubas A. Characterization of charge transfer excited states in [2Fe–2S] iron–sulfur clusters using conventional configuration interaction techniques. Theor. Chem. Acc. 2020, 139, 120 10.1007/s00214-020-02635-7.

[ref138] Presti D.; Stoneburner S. J.; Truhlar D. G.; Gagliardi L. Full Correlation in a Multiconfigurational Study of Bimetallic Clusters: Restricted Active Space Pair-Density Functional Theory Study of [2Fe–2S] Systems. J. Phys. Chem. C 2019, 123, 11899 10.1021/acs.jpcc.9b00222.

[ref174] Almlöf J.; Taylor P. R. Atomic Natural Orbital (ANO) Basis Sets for Quantum Chemical Calculations. Adv. Quantum Chem. 1991, 22, 301–373. 10.1016/S0065-3276(08)60366-4.

[ref139] Veryazov V.; Malmqvist P. A.; Roos B. O. How to select active space for multiconfigurational quantum chemistry?. Int. J. Quantum Chem. 2011, 111, 3329 10.1002/qua.23068.

[ref140] Fulde P. Correlated Electrons in Quantum Matter. World Scientific, 2012. 10.1142/8419.

[ref141] Andersson K.; Roos B. O. Excitation energies in the nickel atom studied with the complete active space SCF method and second-order perturbation theory. Chem. Phys. Lett. 1992, 191, 507 10.1016/0009-2614(92)85581-T.

[ref142] Kramers H. L’interaction Entre les Atomes Magnétogènes dans un Cristal Paramagnétique. Physica 1934, 1, 182 10.1016/S0031-8914(34)90023-9.

[ref143] Anderson P. W. Antiferromagnetism. Theory of Superexchange Interaction. Phys. Rev. 1950, 79, 350 10.1103/PhysRev.79.350.

[ref144] Goodenough J. B. Theory of the Role of Covalence in the Perovskite-Type Manganites [La, *M*(II)]MnO_3_. Phys. Rev. 1955, 100, 564 10.1103/PhysRev.100.564.

[ref145] Kanamori J. Superexchange interaction and symmetry properties of electron orbitals. J. Phys. Chem. Solids 1959, 10, 87 10.1016/0022-3697(59)90061-7.

[ref146] Pipek J.; Mezey P. G. A fast intrinsic localization procedure applicable for ab initio and semiempirical linear combination of atomic orbital wave functions. J. Chem. Phys. 1989, 90, 4916 10.1063/1.456588.

[ref147] Noodleman L.; Baerends E. J. Electronic structure, magnetic properties, ESR, and optical spectra for 2-iron ferredoxin models by LCAO-X.alpha. valence bond theory. J. Am. Chem. Soc. 1984, 106, 2316 10.1021/ja00320a017.

[ref148] Spiller N.; Chilkuri V. G.; DeBeer S.; Neese F. Sulfur vs. Selenium as Bridging Ligand in Di-Iron Complexes: A Theoretical Analysis. Eur. J. Inorg. Chem. 2020, 2020, 1525 10.1002/ejic.202000033.

[ref149] Herrmann C.; Reiher M.; Hess B. A. Comparative analysis of local spin definitions. J. Chem. Phys. 2005, 122, 034102 10.1063/1.1829050. 15740187

[ref150] Ramos-Cordoba E.; Matito E.; Mayer I.; Salvador P. Toward a Unique Definition of the Local Spin. J. Chem. Theory Comput. 2012, 8, 1270 10.1021/ct300050c. 26596743

[ref151] Clark A. E.; Davidson E. R. Local spin. J. Chem. Phys. 2001, 115, 7382 10.1063/1.1407276.

[ref152] Schönemann P. H. A generalized solution of the orthogonal procrustes problem. Psychometrika 1966, 31, 1 10.1007/BF02289451.

[ref153] Malrieu J. P.; Caballol R.; Calzado C. J.; de Graaf C.; Guihéry N. Magnetic Interactions in Molecules and Highly Correlated Materials: Physical Content, Analytical Derivation, and Rigorous Extraction of Magnetic Hamiltonians. Chem. Rev. 2014, 114, 429 10.1021/cr300500z. 24102410

[ref154] de Graaf C.; Broer R. Magnetic Interactions in Molecules and Solids; Springer Int. Pub., 2016.

[ref155] Cabrero J.; Calzado C. J.; Maynau D.; Caballol R.; Malrieu J. P. Metal-Ligand Delocalization in Magnetic Orbitals of Binuclear Complexes. J. Phys. Chem. A 2002, 106, 8146 10.1021/jp0204410.

[ref156] Calzado C. J.; Angeli C.; Taratiel D.; Caballol R.; Malrieu J.-P. Analysis of the magnetic coupling in binuclear systems. III. The role of the ligand to metal charge transfer excitations revisited. J. Chem. Phys. 2009, 131, 044327 10.1063/1.3185506. 19655887

[ref157] Angeli C.; Calzado C. J. The role of the magnetic orbitals in the calculation of the magnetic coupling constants from multireference perturbation theory methods. J. Chem. Phys. 2012, 137, 034104 10.1063/1.4735018. 22830680

[ref158] Levy B.; Berthier G. Generalized brillouin theorem for multiconfigurational SCF theories. Int. J. Quantum Chem. 1968, 2, 307 10.1002/qua.560020210.

[ref159] Calzado C. J.; Cabrero J.; Malrieu J. P.; Caballol R. Analysis of the magnetic coupling in binuclear complexes. I. Physics of the coupling. J. Chem. Phys. 2002, 116, 2728 10.1063/1.1430740. 19655887

[ref160] Calzado C. J.; Cabrero J.; Malrieu J. P.; Caballol R. Analysis of the magnetic coupling in binuclear complexes. II. Derivation of valence effective Hamiltonians from ab initio CI and DFT calculations. J. Chem. Phys. 2002, 116, 3985 10.1063/1.1446024.

[ref161] Broer R.; Maaskant W. Ab initio study of the singlet–triplet splitting in simple models for dichloro- and difluoro-bridged Cu(II) dimers. Chem. Phys. 1986, 102, 103 10.1016/0301-0104(86)85121-7.

[ref162] Miralles J.; Daudey J.-P.; Caballol R. Variational calculation of small energy differences. The singlet-triplet gap in [Cu_2_Cl_6_]^−^]. Chem. Phys. Lett. 1992, 198, 555 10.1016/0009-2614(92)85030-E.

[ref163] Miralles J.; Castell O.; Caballol R.; Malrieu J.-P. Specific CI calculation of energy differences: Transition energies and bond energies. Chem. Phys. 1993, 172, 33 10.1016/0301-0104(93)80104-H.

[ref164] García V. M.; Castell O.; Caballol R.; Malrieu J. An iterative difference-dedicated configuration interaction. Proposal and test studies. Chem. Phys. Lett. 1995, 238, 222 10.1016/0009-2614(95)00438-A.

[ref165] Muñoz D.; Graaf C. D.; Illas F. Putting error bars on the Ab Initio theoretical estimates of the magnetic coupling constants: The parent compounds of superconducting cuprates as a case study. J. Comput. Chem. 2004, 25, 1234 10.1002/jcc.20052. 15139036

[ref166] Jmol: an open-source Java viewer for chemical structures in 3D; http://www.jmol.org/.

[ref167] Galvan I. F. Pegamoid: Orbital viewer for OpenMolcas; https://pypi.org/project/Pegamoid/.

[ref168] Lu T.; Chen F. Multiwfn: A multifunctional wavefunction analyzer. J. Comput. Chem. 2012, 33, 580 10.1002/jcc.22885. 22162017

[ref169] Anderson P. Theory of Magnetic Exchange Interactions:Exchange in Insulators and Semiconductors. In Solid State Physics; Academic Press, 1963; Vol. 14, pp 99–214.

[ref170] Stavrev K. K.; Zerner M. C. Spin-averaged Hartree-Fock procedure for spectroscopic calculations: The absorption spectrum of Mn_2_^+^ in ZnS crystals. Int. J. Quantum Chem. 1997, 65, 877 10.1002/(SICI)1097-461X(1997)65:5<877::AID-QUA51>3.0.CO;2-T.

[ref171] Angeli C. On the nature of the π → π* ionic excited states: The V state of ethene as a prototype. J. Comput. Chem. 2009, 30, 1319 10.1002/jcc.21155. 19009592

[ref172] Paldus J. Many-Electron Correlation Problem A Group Theoretical Approach. In Theoretical Chemistry Advances and Perspectives; Eyring H., Ed.; Elsevier Science, 2012.

[ref173] Pauli W. Über den Zusammenhang des Abschlusses der Elektronengruppen im Atom mit der Komplexstruktur der Spektren. Z. Phys. 1925, 31, 765 10.1007/BF02980631.

