# Supplementary material for: Spin-Pure Stochastic-CASSCF via GUGA-FCIQMC Applied to Iron–Sulfur Clusters
Source: J Chem Theory Comput. 2021 Sep 1;17(9):5684–703. doi: 10.1021/acs.jctc.1c00589 (PMC8444347; doi:10.1021/acs.jctc.1c00589)
Supplement: Supplementary file 1 — ct1c00589_si_001.zip [file ct1c00589_si_001.zip › SupportingInformation.pdf]

Supporting Information: Spin-pure Stochastic-CASSCF via GUGA-FCIQMC applied to Iron Sulfur Clusters

Werner Dobrautz,[a] Oskar Weser, Nikolay Bogdanov, and Giovanni Li Manni
Max Planck Institute for Solid State Research, Heisenbergstr. 1, 70569 Stuttgart, Germany
Ali Alavi
Max Planck Institute for Solid State Research, Heisenbergstr. 1, 70569 Stuttgart, Germany and Yusuf Hamied Department of Chemistry, University of Cambridge, Lensfield Road, Cambridge CB2 1EW, United Kingdom

(Dated: 11 August 2021)

CONTENTS

I. Computational details 1
A. Geometry 1
B. Orbitals 1
C. GUGA-FCIQMC calculation details 2
D. Computational cost 2

II. Comparison with exact results 2

III. Detailed RDM analysis 3

IV. Improved convergence due to stochastic noise 3

V. Wave function statistics for the FeS dimer singlet ground states 3

VI. Orbital comparison protocol 3

VII. Pre-contraction and storage convention of 2-RDMs in `OpenMolcas` 5

VIII. Overview of literature results on the Fe_2S_2 models 6

I. COMPUTATIONAL DETAILS

A. Geometry

The coordinates of the $[Fe_2S_2(SCH_3)_4]^{2-}$ and $[Fe_4S_4(SCH_3)_4)]^{2-}$ models can be found in Table S-I and directly attached in the electronic supporting information (eSI)[1] as files `geometry_Fe2S2.xyz` and `geometry_Fe4S4.xyz` in the `xyz` format.

B. Orbitals

The starting orbitals were obtained by a single-reference high-spin, (10e,10o) $S = 5$ for Fe_2S_2 and (20e,20o) $S = 10$ for Fe_4S_4, ROHF calculation. For

TABLE S I. $[Fe_2S_2(SCH_3)_4]^{2-}$ and $[Fe_4S_4(SCH_3)_4)]^{2-}$ coordinates in Å.

	$[Fe_2S_2(SCH_3)_4]^{2-}$				$[Fe_4S_4(SCH_3)_4)]^{2-}$		
	x	y	z		x	y	z
FE	0.000000	0.000000	1.312783	Fe	0.05	-1.37	1.01
FE	0.000000	0.000000	-1.312783	Fe	-1.38	0.05	-1.00
S	0.757448	-1.576802	0.000000	Fe	-0.05	1.38	1.00
S	-0.757448	1.576802	0.000000	Fe	1.37	-0.05	-1.01
S	1.583457	0.881621	2.761494	S	0.04	-1.78	-1.29
S	-1.583457	-0.881621	2.761494	S	-0.04	1.78	-1.29
S	-1.583457	-0.881621	-2.761494	S	1.78	-0.04	1.29
S	1.583457	0.881621	-2.761494	S	-1.78	0.04	1.29
C	-2.006854	0.653551	3.669027	S	0.24	3.30	2.14
C	2.006854	-0.653551	3.669027	S	-0.24	-3.29	2.14
C	2.006854	-0.653551	-3.669027	S	-3.29	-0.24	-2.14
C	-2.006854	0.653551	-3.669027	S	3.29	0.24	-2.14
H	-2.501125	1.369319	2.995471	C	-3.80	-1.84	-1.38
H	2.501125	-1.369319	2.995471	H	-3.91	-1.71	-0.29
H	2.501125	-1.369319	-2.995471	H	-4.76	-2.17	-1.81
H	-2.501125	1.369319	-2.995471	H	-3.03	-2.60	-1.56
H	-2.683553	0.421356	4.508610	C	3.80	1.83	-1.38
H	2.683553	-0.421356	4.508610	H	3.91	1.71	-0.29
H	2.683553	-0.421356	-4.508610	H	4.76	2.16	-1.81
H	-2.683553	0.421356	-4.508610	H	3.03	2.59	-1.55
H	-1.086873	1.121991	4.050762	C	-1.83	-3.80	1.38
H	1.086873	-1.121991	4.050762	H	-2.16	-4.76	1.81
H	1.086873	-1.121991	-4.050762	H	-2.59	-3.03	1.55
H	-1.086873	1.121991	-4.050762	H	-1.70	-3.91	0.29
				C	1.84	3.80	1.38
				H	2.17	4.76	1.81
				H	2.60	3.03	1.56
				H	1.71	3.91	0.29

Fe_2S_2 we identified the resulting iron 3d and 3d′ orbital and subsequently separately localized them using the Pipek-Mezey[2] method, while the bridging sulfur 3p orbitals were left delocalized. To ensure the most compact description and enable the local spin measurement the resulting orbitals were ordered in the following way: $Fe_A(3d_1\text{-}3d_5)$ $Fe_A(3d'_1\text{-}3d'_5)$ $Fe_B(3d_1\text{-}3d_5)$ $Fe_B(3d'_1\text{-}3d'_5)$ $S_A(3p_1\text{-}3p_3)$ $S_B(3p_1\text{-}3p_3)$

For the Fe_4S_4 system we identified the iron 3d orbitals and performed a Pipek-Mezey localization. The resulting orbitals were ordered atom-by-atom in the following way: $Fe_A(3d_1\text{-}3d_5)$ $Fe_B(3d_1\text{-}3d_5)$ $Fe_C(3d_1\text{-}3d_5)$ $Fe_D(3d_1\text{-}3d_5)$, where the irons A, B and C, D have a farther distance than, e.g. A, C.

The corresponding ROHF starting or-

[a] Electronic mail: w.dobrautz@fkf.mpg.de

TABLE S II. (22e,26o) active space total energies in Hartree (shifted by a -5029.85 H) obtained via the 1- and 2-RDM of the first CASSCF iteration (CASCI) for different spin states as a function of walkers.

N_w	S = 0	S = 1	S = 2
$5 \cdot 10^5$	-0.00805	-0.00602	-0.00360
10^8	-0.00814	-0.00658	-0.00389
$2 \cdot 10^8$	-0.00822	-0.00669	-0.00400
$5 \cdot 10^8$	-0.00829	-0.00685	-0.00414
10^9	-0.00831	-0.00693	-0.00410

bitals are directly attached in the eSI[1] as `fe2s2_22in26_ROHF.orbitals` and `fe4s4_ROHF.orbitals` in the `OpenMolcas RasOrb` format. Additionally we also provide the state-specific relaxed CASSCF orbitals for each spin state for the Fe_2S_2 (22e,26o) active space and the Fe_4S_4 (20e,20o) active space.

C. GUGA-FCIQMC calculation details

For Fe_2S_2 the (10e,10o), (10e,20o) and (22e,16o) active spaces are exactly solvable and thus the GUGA-FCIQMC calculations are rather easy and will not be specified further here. The (22e,26o) active space on the other hand is a challenging task, especially the low-to-intermediate spin states, $S = 0, 1$ and 2. The used number of walker, N_w, is the most important control parameter of a FCIQMC calculation and not only determines the accuracy, but also the computational effort of a calculation. We performed a convergence study of the (22e,26o) energies and the results can be found in Table S-II. The variation in total energy between $5 \cdot 10^8$ an 10^9 walkers is below mH level and thus we conclude the corresponding total energies are converged.

We used the recently implemented precomputed heat bath excitation generator based on[3,4] and adapted it here to be used in conjunction with the GUGA approach and employed a semi-stochastic space of the 50000 most populated CSFs and chose an initiator threshold of $n_{init} = 3$. Additionally, we utilized the histogram-based time-step adaptation[5] with a integration threshold of 99.999%. We ran our calculations on the Max-Planck Computing Cluster `Vortex` using 32 nodes with 40 computing cores each.

For reproducibility we attached the `OpenMolcas` and `NECI`input files for the Fe_2S_2 singlet (22e,26o) Fe_4S_4 singlet (20e,20o) calculations as `fe2s2_22in26_singlet_molcas.input`, `fe2s2_22in26_singlet_fciqmc.input`, `fe4s4_singlet_molcas.input` and `fe4s4_singlet_fciqmc.input` in the eSI[1].

D. Computational cost

For an evaluation of the additional computational cost of the spin-free sampling we measure the time per iteration per walker of a GUGA-FCIQMC cycle, while sampling RDMs, for the later studied iron-sulfur model systems and compare it to standard GUGA-FCIQMC calculation.

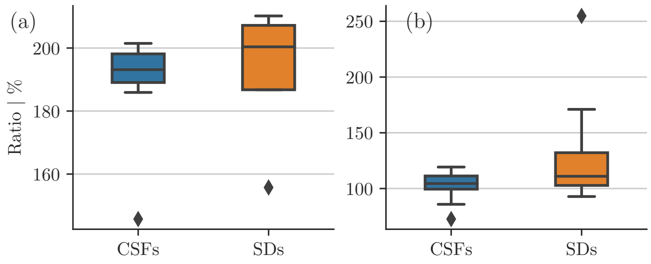

Fig. S 1. Box plot of the additional computational cost of spin-free RDM sampling. (a) Ratio of running a two-replica vs. a single FCIQMC calculation. (b) Overhead due to RDM sampling compared to a two-replica calculation.

Fig. S-1 shows the results of the statistical analysis of time per iteration measurements from multiple runs for several active space sizes and different spin states of the Fe_2S_2 and Fe_4S_4 system. For the statistical analysis, we took the SD- and CSF-based low and high spin calculations of all the 4 studied active spaces of the dimer and of the (20e,20o) active space of the tetramer for into account.

Fig. S-1a shows the ratio of running two replicas in parallel versus a single FCIQMC calculation for SD-based and GUGA-FCIQMC is shown. As expected, there is roughly a two-fold increase in the time per iteration for two replicas, which is a prerequisite for correct RDM sampling[6]. Fig. S-1b shows the additional cost of sampling RDMs compared with a plain two replica calculation. As one can see, apart from one outlier, both the SD- and CSF-based FCIQMC RDM sampling implementations have a marginal computational overhead of 10-20%. The additional communication cost necessary for the spin-free RDM sampling, compared to SDs, is not even noticeable.

In addition to an increased time per iteration, the time-step in the spin-free GUGA-FCIQMC implementation is usually lower compared to the SD-based formulation, which was already studied in Ref. [5]. And although the studied systems here – with almost exclusively open-shell orbitals in the leading terms of the sampled wave function – represents one of the *worst case* situation for a GUGA-based spin-adapted implementation, the effect on the time-step is modest as found in Ref. [5].

II. COMPARISON WITH EXACT RESULTS

To test the validity of the implementation and also analyze the quality of the stochastically sampled RDMs we compared our GUGA-FCIQMC results with FCI results obtained within `Molcas`[7] for the CAS(10e,10o) and CAS(22e,16o) active spaces of the Fe_2S_2 system. Both the CASSCF convergence and direct RDM comparison show the validity and quality of our approach.

Figure S-2 shows the total energy of the $S = 0, 1$ and

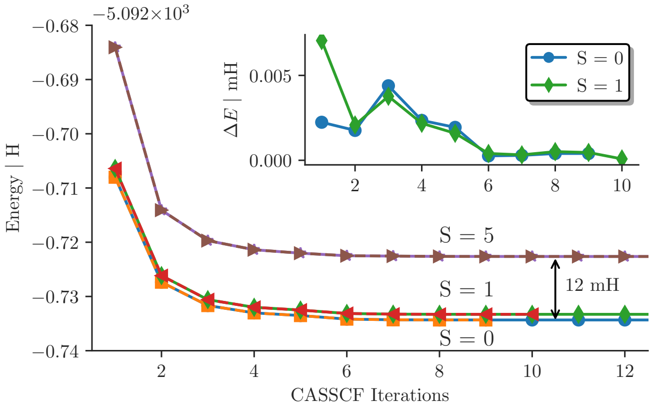

Fig. S 2. Total energy CASSCF results for the $S = 0, 1$ and 5 states of the (10e,10o) active space obtained with GUGA-FCIQMC (dashed lines) compared with exact results (full lines) obtained with `Molcas`[7]. The difference is not noticeable with the eye on this scale, so the inset shows the energy difference of the GUGA-FCIQMC and exact results for the singlet and triplet state.

5 states during a CASSCF calculation obtained with GUGA-FCIQMC as the CI-solver compared to exact results obtained with `Molcas`. For clarity of the figure only those three spin states are shown. All the spin states show excellent agreement with the exact results.

We also explicitly studied the stochastically sampled RDMs, necessary to perform CASSCF. Fig. S-3 shows the 1- and 2-RDM of the singlet state of the (10e,10o) active space obtained exactly from `Molcas` and stochastically with GUGA-FCIQMC. We find excellent agreement in both the one- and two-RDMs, with difference in the order of only 10^{-3} to 10^{-4}, which have a minuscule effect on the energy estimate and CASSCF convergence, as already seen in Fig. S-2

III. DETAILED RDM ANALYSIS

We studied the convergence behavior of the energy obtained via the RDMs and the error (sum of squares, maximum and maximal relative error) of the stochastically sampled RDMs compared to the exact ones as a function of the number of walkers N_w, deterministic space size N_D and sample duration N_S.

In Fig. S-4 we show the Frobenius norm distance, the maximum absolute error, the absolute Hermiticity error and the energy difference between exact and stochastic results for the CAS(10e,10o) singlet state as a function of walkers, semi-stochastic space size and sample durations in a double logarithmic plot. The absolute Hermiticity error is a measure within GUGA-FCIQMC to see how different symmetric RDM entries are due to the stochastic nature of the sampling. On can see in Fig. S-4 the most important influence on it is the size of the semi-stochastic space. For the other accuracy measures all the three parameters, number of walkers, deterministic space size and sample duration have a stark influence. It is important to note that the energy error is orders of magnitudes smaller than the other accuracy measures. This indicates that even a non-perfect RDM sampling in the short term, does not have a hugely negative impact on the energy expectation value. This also means that other properties derived from the 1- and 2-RDMs should have a stable behavior concerning stochastic fluctuations in the sampled wave function.

TABLE S III. CASCI and CASSCF FeS singlet ground state wave function statistics for all considered active spaces in percent. Ref. weight corresponds to the reference weight, MMCT entails all inter-metal charge transfer excitations, Radial d→d' indicates intra-iron "breathing" type excitations, LMCT are bridging-sulfur 3p CT excitations to any iron site and non-Hund stands for intra-iron excitations violating the local Hund's rule.

Active space	10e, 10o		10e, 20o		22e, 16o		22e, 26o	
	casci	casscf	casci	casscf	casci	casscf	casci	casscf
Ref. weight [%]	97.8	**96.5**	90.0	**92.0**	78.4	**61.4**	74.4	**46.1**
MMCT d→d [%]	2.0	**3.1**	6.7	**5.1**	9.6	**13.0**	6.9	**12.9**
Radial d→d'[%]	—	—	1.8	**1.9**	—	—	1.5	**2.1**
LMCT [%]	—	—	—	—	8.5	**19.9**	13.4	**27.9**
non-Hund [%]	0.0	**0.0**	1.3	**0.8**	2.3	**3.7**	1.2	**3.7**

IV. IMPROVED CONVERGENCE DUE TO STOCHASTIC NOISE

For the CAS(10e,20o) with an accidental incorrect starting orbital choice, with iron 4s orbitals in the starting active space instead of two Fe 3d′, there seems to be a positive effect of the stochasticity of the GUGA-FCIQMC RDMs on the CASSCF convergence behavior, as can be seen in Fig. S-5. The *non-optimal* 4s orbitals seem to be expelled from the active space more easily due to the presence of stochastic noise in the RDMs. The end result is, of course, invariant to the choice of the starting orbitals, but apparently stochastic components in the RDMs, in certain situations, help to escape local minima during the CASSCF procedure.

V. WAVE FUNCTION STATISTICS FOR THE FES DIMER SINGLET GROUND STATES

Table S-III contains the explicit values of the wave function contributions to the FeS dimer singlet ground state CASCI and CASSCF results for all considered active spaces.

VI. ORBITAL COMPARISON PROTOCOL

In order to produce the orbital comparison in Fig. M-11 of the main document, we employed the following protocol: The ROHF orbitals were obtained as explained in Sec. I B and used as the starting orbitals for the (22e,26o) CASSCF calculations. The resulting converged singlet (22e,26o) CASSCF orbitals, remained rather localized and the five iron 3d orbitals of iron A are shown in the

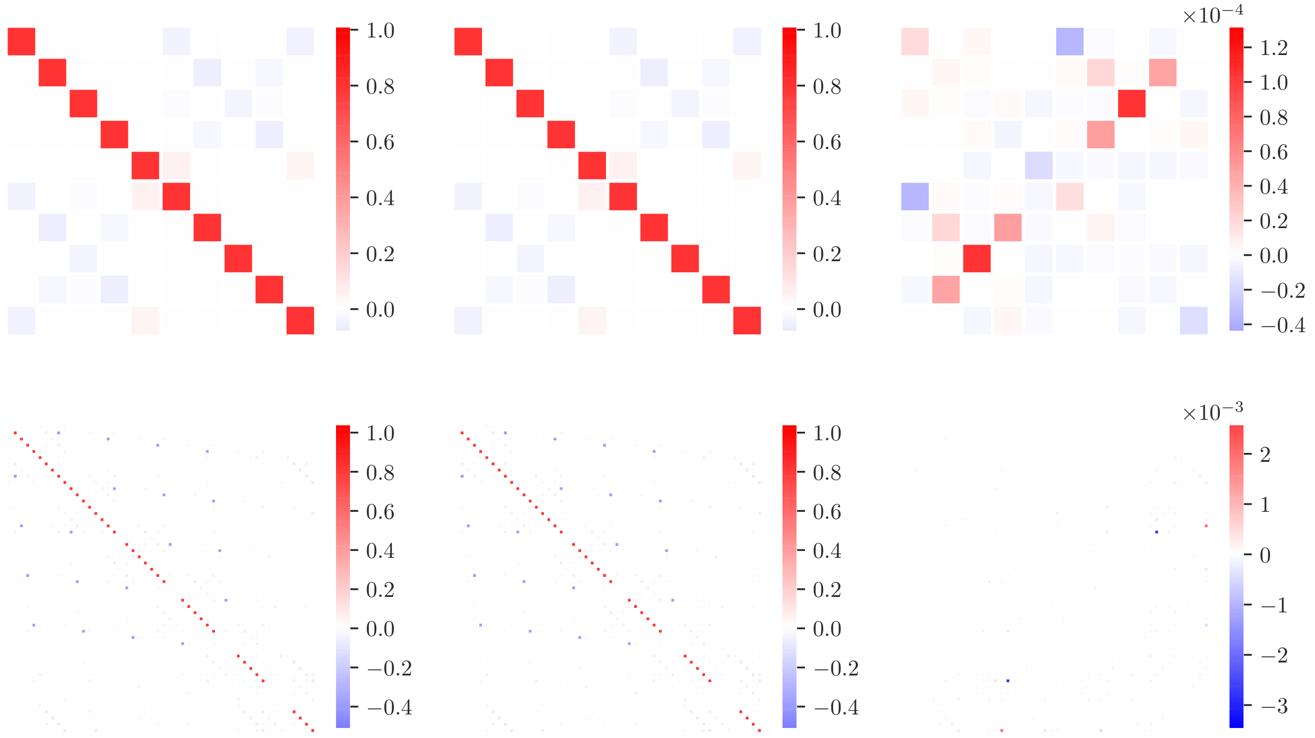

Fig. S 3. (left top and bottom) Exact 1- and 2-RDM obtained within `Molcas`, (middle top and bottom) stochastically sampled 1- and 2-RDM from GUGA-FCIQMC and (right top and bottom) difference for the $S = 0$ CAS(10e, 10o) state. For visibility only the part with the largest errors is shown for the 2-RDM.

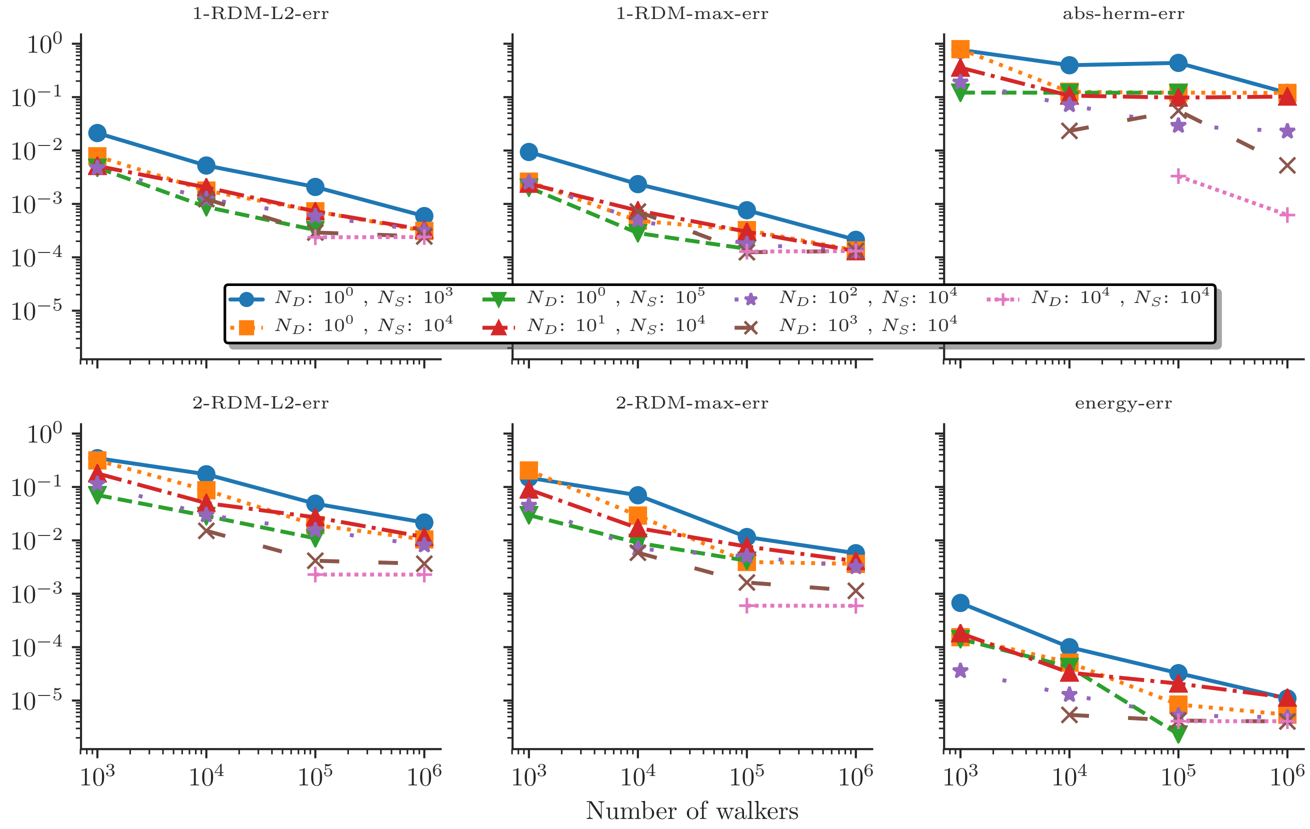

Fig. S 4. Various RDM quality metrics. Explained in the text.

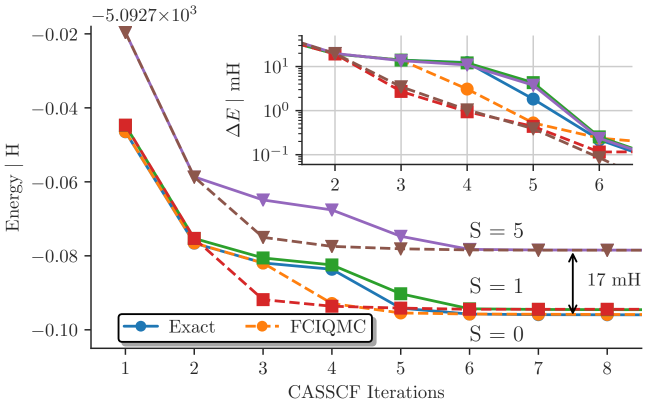

Fig. S 5. Exact (full lines) and stochastic (dashed lines) CASSCF convergence of the (10e, 20o) active space for the $S = 0, 1$ and 5 states. The starting orbitals of this study incorrectly contained iron 4s orbitals in the active space. In this non-optimal scenario the stochastically sampled RDMs seem to have a positive influence on the CASSCF convergence.

second row of Fig. M-11 of the main document with an isosurface value of 0.05.

To enable a clear and direct comparison between the (10e,10o) ROHF and (22e,26o) CASSCF singlet orbitals, the ROHF orbitals were rotated to match the singlet orbitals as closely as possible with the Procrustes algorithm[8,9] within the `OpenMolcas` software package. These rotated (10e,10o) ROHF orbitals are shown in the first row of Fig. M-11 with an isosurface value of 0.05. To compute the difference of these ROHF and CASSCF orbitals, we produced `MOLDEN`[10] files from the corresponding `OpenMolcas` `RasOrb` (before the final conversion to natural orbitals) and used `Pegamoid`[11] to produce `Cube`[12] files with 100 grid points. The `.cub` files were used in `Multiwfn`[13] to calculate the differences, which were visualized with `Jmol`[14], and are shown in the third row of Fig. M-11 of the main document.

VII. PRE-CONTRACTION AND STORAGE CONVENTION OF 2-RDMS IN OPENMOLCAS

For the SCF optimization of orbitals it was necessary to use a format for the two-body RDMs understood by both programs `OpenMolcas` and `NECI`. Since not only `NECI` but also other CI-solvers, based on completely different algorithms (e.g. DMRG), are interfaced to `OpenMolcas`, the details on how the two-body RDMs are stored in `OpenMolcas` may be relevant for other developers as well. For this reason we report the packing, pre-contraction, and internal storage of two-body RDMs in `OpenMolcas`.

As written in the main text, the spin-free one- and two-body RDMs are defined as (following the convention of Helgaker, Jørgensen and Olsen[15])

$$\rho_{ij} = \langle \Psi | \hat{E}_{ij} | \Psi \rangle = \sum_{\mu\nu} c^*_\mu c_\nu \langle \mu | \hat{E}_{ij} | \nu \rangle, \tag{1}$$

with $\hat{E}^\dagger_{ij} = \hat{E}_{ji}$, and

$$\begin{aligned} \Gamma_{ij,kl} &= \langle \Psi | \hat{e}_{ij,kl} | \Psi \rangle = \sum_{\mu\nu} c^*_\mu c_\nu \langle \mu | \hat{e}_{ij,kl} | \nu \rangle \\ &= \sum_{\mu\nu} c^*_\mu c_\nu \langle \mu | \hat{E}_{ij} \hat{E}_{kl} - \delta_{jk} \hat{E}_{il} | \nu \rangle, \end{aligned} \tag{2}$$

with i, j, k, l denoting *spatial* orbitals, $|\mu\rangle$ and $|\nu\rangle$ being configuration state functions (CSFs) and c_μ and c_ν their coefficients in the wave function expansion, $|\Psi\rangle$. The spin-free 2-RDMs exhibit the following 4-fold permutational symmetry

$$\Gamma_{ij,kl} = \Gamma_{kl,ij} = \Gamma^\dagger_{ji,lk} = \Gamma^\dagger_{lk,ji} \quad . \tag{3}$$

The last two equalities reduce to a "normal" equality if only real orbitals are used, which we assume in the following.

Since the calculation of properties often involves a multiplication of Γ with other tensors of higher permutational symmetry with subsequent contraction we can split Γ into a symmetrized and anti-symmetrized part to reduce the operation count. One example is the calculation of the total energy using the reduced density matrices and one and two electron integrals. In line with the literature[15] we define the two electron integral as

$$g_{ijkl} = \iint \phi^\dagger_i(x_1) \phi^\dagger_k(x_2) \frac{1}{r_{1,2}} \phi_j(x_1) \phi_l(x_2) \, \mathrm{d}x_1 \mathrm{d}x_2 \quad . \tag{4}$$

Note that g has the 8-fold symmetry:

$$\begin{aligned} g_{ij,kl} &= g_{kl,ij} = g_{ji,lk} = g_{lk,ji} \\ &= g_{ji,kl} = g_{lk,ij} = g_{ij,lk} = g_{kl,ji} \quad . \end{aligned} \tag{5}$$

The two-electron contribution to the energy is given by a multiplication of Γ and g with subsequent contraction as in the following equation

$$\begin{aligned} \frac{1}{2} \sum_{ijkl} g_{ijkl} \Gamma_{ij,kl} &= \frac{1}{4} \sum_{ijkl} g_{ijkl} \Gamma_{ij,kl} + \sum_{ijkl} g_{jikl} \Gamma_{ji,kl} \\ &= \frac{1}{2} \sum_{ijkl} g_{ijkl} \frac{\Gamma_{ij,kl} + \Gamma_{ji,kl}}{2} \\ &= \frac{1}{2} \sum_{ijkl} g_{ijkl} \, {}^{+}\Gamma_{ij,kl} \quad . \end{aligned} \tag{6}$$

Here we introduced the (anti)-symmetrized 2-RDM

$${}^{\pm}\Gamma_{ij,kl} = \frac{\Gamma_{ij,kl} \pm \Gamma_{ji,kl}}{2} \quad . \tag{7}$$

With equation (3) it can proven that ${}^{\pm}\Gamma$ has the following eightfold symmetries (again assuming real orbitals):

$$\begin{aligned} {}^{\pm}\Gamma_{ij,kl} &= {}^{\pm}\Gamma_{kl,ij} = {}^{\pm}\Gamma_{ji,lk} = {}^{\pm}\Gamma_{lk,ji} \\ &= \pm{}^{\pm}\Gamma_{ji,kl} = \pm{}^{\pm}\Gamma_{lk,ij} = \pm{}^{\pm}\Gamma_{ij,lk} = \pm{}^{\pm}\Gamma_{kl,ji} \, , \end{aligned} \tag{8}$$

which are four more equalities than the original Γ, see Eq. (3)). The joint 8-fold symmetries of ${}^{+}\Gamma$ and g allow a great reduction of operation count in the summation

of the two electron term in Eq. (6).

Using the (anti)-symmetry of ${}^{\pm}\Gamma$ we can store only those elements with $i \geq j$ and $k \geq l$ and use this symmetry to switch from the 4-indexed tensor ${}^{\pm}\Gamma$ to a two-dimensional matrix ${}^{\pm}d$. To do so, we introduce the joint indices

$$\underline{ij} = \begin{cases} \frac{i(i-1)}{2} + j & \text{for} \quad i \geq j \\ \frac{j(j-1)}{2} + i & \text{for} \quad i < j \end{cases}$$
$$\underline{kl} = \begin{cases} \frac{k(k-1)}{2} + l & \text{for} \quad k \geq l \\ \frac{l(l-1)}{2} + k & \text{for} \quad k < l. \end{cases} \tag{9}$$

To account for degeneracy with respect to "Hermitian symmetry" ($\Gamma_{ij,kl} = \Gamma^{\dagger}_{lk,ji}$), we also define

$$n(\underline{kl}) = \begin{cases} 1 & \text{for} \quad k = l \\ 2 & \text{for} \quad k > l. \end{cases} \tag{10}$$

This allows us finally to define

$${}^{\pm}d_{\underline{ij},\underline{kl}} = \frac{1}{2} \cdot n(\underline{kl}) \cdot {}^{\pm}\Gamma_{ij,kl} = \pm\frac{1}{2} \cdot n(\underline{kl}) \cdot {}^{\pm}\Gamma_{ji,kl} \quad . \tag{11}$$

The additional factor of $\frac{1}{2}$ in Eq. (11) is introduced, because the calculation of the total energy requires a multiplication with $\frac{1}{2}$, which can be omitted, if it is contained in the RDM. From Eq. (3) we see that the two-dimensional matrix ${}^{\pm}d$ is symmetric with respect to the joint index, so we have

$${}^{\pm}d_{\underline{ij},\underline{kl}} = {}^{\pm} d_{\underline{kl},\underline{ij}} \ . \tag{12}$$

We can write now explicitly (omitting the redundant upper triangular of ${}^{\pm}d$):

$${}^{\pm}d = \frac{1}{2}\begin{pmatrix} \frac{\Gamma_{11,11}\pm\Gamma_{11,11}}{2} & & & \\ \frac{\Gamma_{21,11}\pm\Gamma_{12,11}}{2} & 2\frac{\Gamma_{21,21}\pm\Gamma_{12,21}}{2} & & \\ \frac{\Gamma_{22,11}\pm\Gamma_{22,11}}{2} & 2\frac{\Gamma_{22,21}\pm\Gamma_{22,21}}{2} & \frac{\Gamma_{22,22}\pm\Gamma_{22,22}}{2} & \\ \frac{\Gamma_{31,11}\pm\Gamma_{13,11}}{2} & 2\frac{\Gamma_{31,21}\pm\Gamma_{13,21}}{2} & \frac{\Gamma_{31,22}\pm\Gamma_{13,22}}{2} & \cdots \\ \frac{\Gamma_{32,11}\pm\Gamma_{23,11}}{2} & 2\frac{\Gamma_{32,21}\pm\Gamma_{23,21}}{2} & \frac{\Gamma_{32,22}\pm\Gamma_{23,22}}{2} & \\ \vdots & & & \ddots \end{pmatrix}$$
$${}^{+}d = \begin{pmatrix} \frac{\Gamma_{11,11}}{2} & & & \\ \frac{\Gamma_{21,11}}{2} & \frac{\Gamma_{21,21}+\Gamma_{12,21}}{2} & & \\ \frac{\Gamma_{22,11}}{2} & \Gamma_{22,21} & \frac{\Gamma_{22,22}}{2} & \\ \frac{\Gamma_{31,11}}{2} & \frac{\Gamma_{31,21}+\Gamma_{13,21}}{2} & \frac{\Gamma_{31,22}}{2} & \cdots \\ \frac{\Gamma_{32,11}}{2} & \frac{\Gamma_{32,21}+\Gamma_{23,21}}{2} & \frac{\Gamma_{32,22}}{2} & \\ \vdots & & & \ddots \end{pmatrix}$$
$${}^{-}d = \begin{pmatrix} 0 & & & \\ 0 & \frac{\Gamma_{21,21}-\Gamma_{12,21}}{2} & & \\ 0 & 0 & 0 & \\ 0 & \frac{\Gamma_{31,21}-\Gamma_{13,21}}{2} & 0 & \cdots \\ 0 & \frac{\Gamma_{32,21}-\Gamma_{23,21}}{2} & 0 & \\ \vdots & & & \ddots \end{pmatrix} \tag{13}$$

The (anti)-symmetrized matrices ${}^{-}d$ and ${}^{+}d$ are called `PAMAT` and `PSMAT` respectively in the code of OpenMolcas. To account for symmetry, only the lower triangular part of ${}^{-}d$ and ${}^{+}d$ is stored as linear vector with the joint index

$$\underline{ijkl} = \begin{cases} \frac{\underline{ij}(\underline{ij}-1)}{2} + \underline{kl} & \underline{ij} \geq \underline{kl} \\ \frac{\underline{kl}(\underline{kl}-1)}{2} + \underline{ij} & \underline{kl} < \underline{kl} \end{cases} \quad . \tag{14}$$

In this (anti)-symmetrized and linearized shape the two-body RDMs are actually stored in `OpenMolcas`.

VIII. OVERVIEW OF LITERATURE RESULTS ON THE FE_2S_2 MODELS

In Table S-IV, we gather the bilinear J and biquadratic, J' and K values and their associated root-mean-square (RMSE) and average relative errors ω along with the employed geometry, active space and basis set level from various computational studies, including DMRG CI-only,[16] BS-DFT,[17,18] N-electron valence state perturbation theory (NEVPT2),[19,20] and restricted active space pair-density functional theory (RAS-PDFT),[21] as well as reference values from a fit to experimental magnetic susceptibility measurements of a similar synthetic dimer.[22]

The CASSCF(10e,10o) calculations, performed by Chilkuri *et al.*,[19] with $J_{CASSCF} = 0.63$ mH, agree rather well with our corresponding result, $J_{10e,10o} = 0.82$ mH. Their slightly smaller value compared to our CASSCF(10e,10o) result, can be related to the slight difference in the studied geometry, as our geometry exhibits a smaller Fe-Fe distance, see Table S-IV. A similar effect of comparable magnitude is found by Spiller *et al.*[20] when comparing extracted magnetic couplings between an optimized and symmetrized geometry, see the SI of Reference [20], where a reduction of the iron-iron distance by 0.02 Å yields an increase of J by ≈ 0.13 mH using the CAS(10e,10o).

With the present very limited set of results no clear trend of the basis set size dependence can be identified. However, studies on computational estimates for magnetic couplings in have shown only a modest basis set dependence.[23–25]

The good quantitative agreement of both our CASCI(22,26) and CASSCF(22,26) results with experimental values of J, see Table S-IV, should be considered cautiously. In fact, (a) our studied geometry slightly differs from the experimental one,[22] (b) only intra-molecular magnetic interactions are considered, while inter-molecular interactions and other environmental effects are ignored in our model, (c) we did not extrapolate our results to the complete basis set limit and, (d) dynamic correlation effects outside the active space are missing.

[1]The Supporting Information can be found at `http://pubs.acs.org`. 2021.
[2]Pipek, J.; Mezey, P. G. A fast intrinsic localization procedure applicable for ab initio and semiempirical linear combination of atomic orbital wave functions. *J. Chem. Phys.* **1989**, *90*, 4916.

TABLE S IV. Magnetic coupling parameter results, J, J' and K in mH of multiple computational studies,[16,17,19–21] experimental results,[22] and the present work (CASSCF results in bold font, CASCI results in normal font). Except for the BS-DFT[17] and the experimental study[22] the model complex $[Fe_2S_2(SCH_3)_4]^{2-}$ was used in all computations. We document the used active spaces (where applicable), geometry (iron-iron, Fe-Fe, and iron-bridging sulfur, Fe-S*, distance in Å, and $\measuredangle$(Fe-S*-Fe)angle in degree) and the basis set order of the iron 3d orbitals. To judge the quality of the fit to a (biquadratic) Heisenberg model, we present the relative ω and of the bilinear (J) and of the biquadratic ($J' + K$) mapping.

Method	GUGA-FCIQMC		DMRG-CI	RASCI	RAS-PDFT		CASSCF	NEVPT2	NEVPT2	BS-DFT	Exp.
Reference	present work		[16]	[21]			[19]		[20]	[17]	[22]
Active space	10e, 10o	22e, 26o	30e, 32o	22e, 21o		22e, 26o	10e, 10o		22e, 16o	–	–
Fe-Fe [Å]	2.63		2.69		2.69		2.69		2.82	2.69	2.70
Fe-S [Å]	2.19		2.20		2.20		2.20		2.22	2.21	2.21
$\measuredangle$(Fe-S*-Fe) [°]	73.8		75.4		75.1		75.3		79.1	75.0	75.3
Fe basis set order	DZ		SVP		TZ		TZ	SVP	TZ	TZ	–
J [mH]	**0.82** / 0.55	**2.45** / 1.44	2.15	1.21	2.29	2.46	0.63	1.02	1.68	2.82	1.35±0.15
J' [mH]	**0.74** / 0.55	**2.70** / 1.47	2.52	1.25	2.42	2.60	0.62	1.01	1.54	–	–
K [mH]	**-0.018** / 0.00	**0.054** / 0.007	0.09	0.009	0.029	0.03	-0.001	-0.002	-0.03	–	–
RMSE(J) [mH]	**0.53** / 0.01	**1.66** / 0.20	2.82	0.26	0.86	0.9	0.04	0.06	0.94	–	–
RMSE($J'+K$) [mH]	**0.02** / 0.00	**0.31** / 0.05	0.87	0.06	0.029	0.03	0.03	0.01	0.23	–	–
$\omega(J)$ [%]	**4.26** / 0.13	**3.89** / 0.81	7.1	1.34	2.29	2.18	0.31	0.38	3.63	–	–
$\omega(J'+K)$ [%]	**0.14** / 0.02	**0.73** / 0.16	2.2	0.25	0.27	0.29	0.25	0.08	0.86	–	–

[3] Holmes, A. A.; Changlani, H. J.; Umrigar, C. J. Efficient Heat-Bath Sampling in Fock Space. *J. Chem. Theory Comput.* **2016**, *12*, 1561.

[4] Guther, K.; Anderson, R. J.; Blunt, N. S.; Bogdanov, N. A.; Cleland, D.; Dattani, N.; Dobrautz, W.; Ghanem, K.; Jeszenszki, P.; Liebermann, N.; Li Manni, G.; Lozovoi, A. Y.; Luo, H.; Ma, D.; Merz, F.; Overy, C.; Rampp, M.; Samanta, P. K.; Schwarz, L. R.; Shepherd, J. J.; Smart, S. D.; Vitale, E.; Weser, O.; Booth, G. H.; Alavi, A. NECI: N-Electron Configuration Interaction with an emphasis on state-of-the-art stochastic methods. *J. Chem. Phys.* **2020**, *153*, 034107.

[5] Dobrautz, W.; Smart, S. D.; Alavi, A. Efficient formulation of full configuration interaction quantum Monte Carlo in a spin eigenbasis via the graphical unitary group approach. *J. Chem. Phys.* **2019**, *151*, 094104.

[6] Overy, C.; Booth, G. H.; Blunt, N. S.; Shepherd, J. J.; Cleland, D.; Alavi, A. Unbiased reduced density matrices and electronic properties from full configuration interaction quantum Monte Carlo. *J. Chem. Phys.* **2014**, *141*, 244117.

[7] Aquilante, F.; Autschbach, J.; Carlson, R. K.; Chibotaru, L. F.; Delcey, M. G.; De Vico, L.; Fdez. Galván, I.; Ferré, N.; Frutos, L. M.; Gagliardi, L.; Garavelli, M.; Giussani, A.; Hoyer, C. E.; Li Manni, G.; Lischka, H.; Ma, D.; Malmqvist, P.-Å.; Müller, T.; Nenov, A.; Olivucci, M.; Pedersen, T. B.; Peng, D.; Plasser, F.; Pritchard, B.; Reiher, M.; Rivalta, I.; Schapiro, I.; Segarra-Martí, J.; Stenrup, M.; Truhlar, D. G.; Ungur, L.; Valentini, A.; Vancoillie, S.; Veryazov, V.; Vysotskiy, V. P.; Weingart, O.; Zapata, F.; Lindh, R. Molcas 8: New capabilities for multiconfigurational quantum chemical calculations across the periodic table. *J. Comput. Chem.* **2016**, *37*, 506.

[8] Schönemann, P. H. A generalized solution of the orthogonal procrustes problem. *Psychometrika* **1966**, *31*, 1.

[9] Weser, O.; Freitag, L.; Guther, K.; Alavi, A.; Li Manni, G. Chemical insights into the electronic structure of Fe(II) porphyrin using FCIQMC, DMRG, and generalized active spaces. *Int. J. Quantum Chem.* **2021**, *121*, e26454.

[10] Schaftenaar, G.; Noordik, J. Molden: a pre- and post-processing program for molecular and electronic structures. *J. Comput.-Aided Mol. Des.* **2000**, *14*, 123.

[11] Galvan, I. F. Pegamoid: Orbital viewer for OpenMolcas. `https://pypi.org/project/Pegamoid/`.

[12] Gaussian Cube Files. `http://paulbourke.net/dataformats/cube/`.

[13] Lu, T.; Chen, F. Multiwfn: A multifunctional wavefunction analyzer. *J. Comput. Chem.* **2011**, *33*, 580.

[14] Jmol: an open-source Java viewer for chemical structures in 3D. `http://www.jmol.org/`.

[15] Helgaker, T.; Jørgensen, P.; Olsen, J. *Molecular Electronic-Structure Theory*; John Wiley & Sons: Chichester, 2000.

[16] Sharma, S.; Sivalingam, K.; Neese, F.; Chan, G. K.-L. Low-energy spectrum of iron–sulfur clusters directly from many-particle quantum mechanics. *Nat. Chem.* **2014**, *6*, 927.

[17] Noodleman, L.; Baerends, E. J. Electronic structure, magnetic properties, ESR, and optical spectra for 2-iron ferredoxin models by LCAO-X.alpha. valence bond theory. *J. Am. Chem. Soc.* **1984**, *106*, 2316.

[18] Noodleman, L.; Case, D. A. In *Density-Functional Theory of Spin Polarization and Spin Coupling in Iron—Sulfur Clusters*; Cammack, R., Ed.; Advances in Inorganic Chemistry; Academic Press, 1992; Vol. 38; p 423.

[19] Chilkuri, V. G.; DeBeer, S.; Neese, F. Ligand Field Theory and Angular Overlap Model Based Analysis of the Electronic Structure of Homovalent Iron–Sulfur Dimers. *Inorg. Chem.* **2019**, *59*, 984.

[20] Spiller, N.; Chilkuri, V. G.; DeBeer, S.; Neese, F. Sulfur vs. Selenium as Bridging Ligand in Di-Iron Complexes: A Theoretical Analysis. *Eur. J. Inorg. Chem.* **2020**, *2020*, 1525.

[21] Presti, D.; Stoneburner, S. J.; Truhlar, D. G.; Gagliardi, L. Full Correlation in a Multiconfigurational Study of Bimetallic Clusters: Restricted Active Space Pair-Density Functional Theory Study of [2Fe–2S] Systems. *J. Phys. Chem. C* **2019**, *123*, 11899.

[22] Gillum, W. O.; Frankel, R. B.; Foner, S.; Holm, R. H. Synthetic analogues of the active sites of iron-sulfur proteins. XIII. Further electronic structural relationships between the analogues $[Fe_2S_2(SR)_4]^{2-}$ and the active sites of oxidized 2Fe-2S proteins. *Inorg. Chem.* **1976**, *15*, 1095.

[23] Muñoz, D.; Graaf, C. D.; Illas, F. Putting error bars on the *Ab Initio* theoretical estimates of the magnetic coupling constants: The parent compounds of superconducting cuprates as a case study. *J. Comput. Chem.* **2004**, *25*, 1234.

[24] Queralt, N.; de Graaf, C.; Cabrero, J.; Caballol, R. Ferrimagnetic coupling in oxamido-bridged Mn(II)Cu(II) compounds: a combined CASPT2 and DDCI study. *Mol. Phys.* **2003**, *101*, 2095.

[25] Casanovas, J.; Illas, F. An ab initio cluster model study of the magnetic coupling in KNiF3. *J. Chem. Phys.* **1994**, *100*, 8257.
